# Pink noise reduces impact of traffic noise on sleep and the blood metabolome: a cross-over pilot study

**DOI:** 10.1038/s43856-026-01380-5

**Published:** 2026-01-10

**Authors:** Natalia Vincens, Anna Nause, Mathias Basner, Sofie Fredriksson, Daniel Malmodin, Anders Bay Nord, Kerstin Persson Waye, Magdy Younes, Ding Zou, Michael G. Smith

**Affiliations:** 1 School of Public Health and Community Medicine, Institute of Medicine, Sahlgrenska Academy, University of Gothenburg, Gothenburg, Sweden; 2 Unit for Experimental Psychiatry, Division of Sleep and Chronobiology, Department of Psychiatry, University of Pennsylvania Perelman School of Medicine, Philadelphia, PA USA; 3 Region Västra Götaland, Habilitation and Health, Hearing Organization, Gothenburg, Sweden; 4 National Bioinformatics Infrastructure Sweden (NBIS), Gothenburg, Sweden; 5 Swedish NMR Centre, University of Gothenburg, Gothenburg, Sweden; 6 Sleep Disorders Centre, University of Manitoba, Winnipeg, MB Canada; 7 Center for Sleep and Vigilance Disorders, Sahlgrenska Academy, University of Gothenburg, Gothenburg, Sweden

**Keywords:** Predictive markers, Metabolic disorders, Biomarkers, Medical research

## Abstract

**Background:**

Epidemiological studies show associations between chronic noise exposure and disease, but the biological pathways remain poorly understood. In this explorative pilot study, we investigate the mechanisms that may link sleep disruption by environmental noise with disease, and the efficacy of a non-pharmacological intervention to mitigate these effects.

**Methods:**

We conducted a cross-over trial (ClinicalTrials.gov: NCT05319262; 2022-03-09) where N = 12 healthy individuals slept for five consecutive nights in acoustically isolated bedrooms. Nights included one familiarisation night; one quiet baseline night; one night with road, rail and air traffic noise of levels 45-65 dB *L*_AS,max_; one night with continuous 45 dB broadband pink noise; and one night with both traffic noise and pink noise. Sleep was measured with polysomnography. Perceived sleep quality and recouperation were measured with morning questionnaires. Daily blood samples were collected for metabolomics analysis.

**Results:**

Here we show that discrete traffic noise events induced acute elevations of the odds ratio product, indicating acute sleep fragmentation, even while total sleep time and overall sleep macrostructure were preserved. Traffic noise is further associated with significant elevations in concentrations of leucine, lactic acid, and acetone relative to quiet control. Sleep and metabolic disturbances by traffic noise are attenuated when pink noise is played continuously throughout the night.

**Conclusions:**

Noise-induced sleep fragmentation is followed by changes in metabolic processes that in the long-term may be precursors for cardiometabolic disorders. Masking of traffic noise by continuous, neutral sound may mitigate acute physiological sleep disturbance and downstream metabolic effects. These results should be interpreted cautiously, given the limited sample size and subject homogeneity.

## Plain Language Summary

This study explored how environmental noise at night might affect health and whether a simple sound-based intervention could help. Twelve healthy adults slept under different sound conditions, including quiet, traffic noise, and continuous low volume noise, termed “pink noise”. Researchers measured sleep quality and changes in certain compounds in the blood. Traffic noise caused brief disruptions in sleep and altered levels of certain compounds linked to energy use. However, during continuous playback of pink noise, these negative effects were reduced. These findings suggest that even when total sleep time is not affected, noise can disturb the body’s physiology in ways that may affect long-term health. Continuous neutral sounds like pink noise could help protect sleep and well-being in noisy environments.

## Abstract (web summary)

Vincens et al. investigate how traffic noise and masking pink noise impact physiological sleep, cognition, and metabolic blood biomarkers. Traffic noise acutely fragments sleep even while total sleep time is preserved, and is followed by metabolic disturbances, the effects of which are attenuated by continuous pink noise.

## Introduction

Environmental noise is a widely prevalent stressor. In terms of environmental factors that contribute to disease burden, traffic noise is second only to, and in some high-income countries comparable to, air pollution^1^. Cross-sectional epidemiological studies repeatedly demonstrate associations between traffic noise and multiple cardiometabolic outcomes, which include ischaemic heart disease, hypertension, stroke, diabetes and obesity^2,3^. Associations are strongest for night-time levels, implicating sleep disturbance as a key pathway between noise and disease^4,5^. Sleep is a vital physiological process that has critical roles in maintaining overall health. Disruption of sleep is accordingly linked with increased risks for various chronic diseases, including cardiometabolic disorders and diseases^6,7^. Despite the associations between noise exposure and health effects, the mechanisms underlying the relationship between noise, sleep, and cardiometabolic disorders remain poorly understood.

The noise reaction scheme proposes that noise exposure at relatively low levels can interfere with concentration, relaxation or sleep^8^. This could happen directly, through sound levels activating sympathetic response or release of corticosteroids, or indirectly, via the emotional and subjective perception of the sound. These stress responses involve brain regions that have inputs to the autonomic nervous system, endocrine system and limbic system. Such responses can trigger acute cardiovascular effects, including increases in stress hormones and endothelial dysfunction, as shown in healthy volunteers exposed to simulated night-time transportation noise^9,10^, and discussed by Münzel et al. in a comprehensive review on epidemiological as well as experimental research^11^. Short-term nocturnal transportation noise has also been found to impair glucose regulation, with higher post-load glucose and insulin levels observed after four nights of noise exposure, suggesting transient insulin resistance even in the absence of disturbance in sleep macrostructure^12^. Since stress and sleep disruption pathways intersect with core metabolic processes, metabolomics can provide a suitable approach to capture downstream biochemical changes, like the ones related to sleep disruption^13,14^, but specifically associated with nighttime noise exposure, such as alterations in lipid, amino acid, and energy metabolism^9,10,12^. Metabolites are attractive as biomarkers of cardiometabolic disorders since they are involved in disease pathways that lead to their accumulation or deficiency^15^. By providing endpoints or intermediaries of metabolic processes, and thus providing insights into multiple aspects of cellular physiology, metabolomics can offer an understanding of the underlying mechanisms linking disease with nocturnal noise via sleep fragmentation or even other stress-related pathways^12,16–18^.

So-called “white noise machines” are non-pharmacological sleep aids that continue to grow in popularity. The broadband sound they emit may actively promote sleep, mask the presence of environmental noises that would otherwise disrupt sleep, or act as a stimulus control when used as part of a regular sleep routine^19^. Although overall evidence for the efficacy of continuous noise to improve sleep remains mixed and of very low quality^19^, the masking of disruptive sounds is mechanistically credible^20^. If sleep disturbance by traffic noise is a direct cause of biological changes relevant to cardiometabolic health, then mitigating the effects of noise on sleep with broadband masking sound should attenuate these changes. Further, the potential adverse effects of broadband sound as a sleep aid remain unclear.

We here perform an experimental laboratory study to address the knowledge gap regarding the metabolic effects of nocturnal noise exposure in humans. Specifically, in this exploratory pilot study, we aim to determine if acute sleep fragmentation by traffic noise is associated with metabolic changes that support epidemiological associations with adverse cardiometabolic health, and if these changes are mitigated by pink noise. Data show that nights with acutely sleep-disturbing noise are followed by changes in markers of metabolic processes. Masking the sleep-disturbing noise with broadband pink noise attenuates both the physiological sleep disturbance and the metabolic changes. These mechanistic insights may help explain epidemiological relationships between environmental stressors during sleep and chronic disease.

## Methods

The study was approved by the local ethics committee (Swedish Ethical Review Authority, 2021-06812-01). The study protocol was registered prior to subject recruitment on ClinicalTrials.gov (NCT05319262, 2022-03-09). Study subjects provided informed consent prior to the start of the study, were financially compensated for their participation, and could discontinue at any time without explanation. There was no patient or public involvement in the design, conduct or reporting of the study, except that study participants were recruited via public advertising.

### Design

The experiment was performed in the sound environment laboratory (SEL) at the University of Gothenburg from May to June 2022. The SEL is a high-fidelity research laboratory equipped to simulate a typical apartment, including three individually light-, sound- and vibration-isolated private bedrooms.

**Fig. 1:**
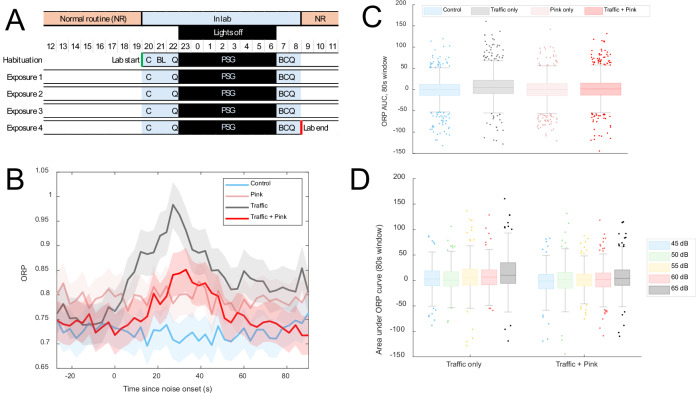
Experimental study of sleep fragmentation by traffic noise and masking by pink noise. **A** Study protocol. Data collection is indicated as follows C Cognition; BL Baseline questionnaires; Q Daily questionnaires; PSG Polysomnography; B Blood draws. The first night was for habituation to study setting and procedures. The following exposure nights 1–4 were randomised order: Control (quiet), Traffic noise, Pink noise, and Traffic + Pink noise. **B** Event-related change in ORP stratified by experimental condition. Data are epoch-by-epoch mean values across all noise events and study subjects. Shaded areas indicate 95% confidence intervals. **C** Boxplots for event-related area under the ORP curve. *N* = 1320 for each of the four conditions (total *N* = 5280). **D** Boxplots of exposure-response relationships for event-related area under the curve) odds ratio product (ORP) in nights with traffic noise *N* = 264 for each of the five noise levels in each exposure condition (total *N* = 2640).

The study used a prospective within-subjects cross-over design (Fig. 1A). Participants spent five consecutive nights in the SEL, with a scheduled sleep opportunity between 23:00-07:00. The first night was a habituation period to the study protocol and for familiarisation with the test procedures. Study nights 2–5 were experimental nights and were randomly assigned (by investigator M.G.S.) across participants using a Latin square design to avoid first-order carryover effects. Randomisation was done by investigator M.G.S. using a decentralised computer. Study participants and other investigators were blinded to the upcoming exposure condition. Data analysts were blinded to the exposure condition via coding of nights as a randomly generated number.

Each subject was exposed to one night of each of the following:

Quiet night: No noise was played. This night served as a control night to assess individual baseline sleep, metabolic profile, and cognitive performance.

Traffic noise at night: to determine the consequences of noise-disrupted sleep;

Pink noise night: to determine the impact of continuous pink noise on sleep;

Traffic + pink noise night: simultaneous traffic and pink noise at the same sound pressure levels as in the traffic-only and pink noise-only nights, to determine sleep-protecting effects of pink noise in the face of traffic noise.

Subjects could follow their normal daytime routine, but were prohibited from alcohol, ingesting caffeine after 15:00, and napping. Abstaining from napping was confirmed with measures of daytime activity via wrist actigraphy monitors (ActiGraph wGT3x-BT, Pensacola, FL) worn continuously throughout the study. Subjects arrived at the SEL by 20:00 each evening to complete cognitive testing. They were then hooked up to the sleep measurement apparatus one by one. Subjects were instructed to turn off the lights and start trying to sleep at 23:00. They were woken each morning at 07:00 by an alarm call broadcast into the bedroom. They then provided a blood sample and completed questionnaires and cognitive testing, after which they followed their normal routine until returning to the SEL the following evening.

### Noise exposures

Noise was introduced via ceiling-mounted speakers in each bedroom. All sound pressure levels were calibrated to 10 cm above the pillow in each bedroom prior to the study, so that these levels accurately reflect the noise exposure of the subjects during sleep. Noise levels in the different study nights are summarised in Table 1. In the quiet control night, no traffic noise or pink noise was introduced. Because the background levels in the SEL were unnaturally quiet ( < 13 dB) even with the ventilation system turned on, we artificially introduced low-level (20 dB) background ventilation noise via the audio system. This low level does not affect sleep, yet introducing it contributes to a more naturalistic bedroom environment.

**Table 1:** Noise exposures in each experimental condition

Night	*L*_night_	*L*_AS,max,traffic_
Habituation/control	20 dB	-
Traffic	43.6 dB	65 dB
Pink	45.0 dB	-
Traffic + Pink	47.0 dB	65 dB

*L*_night_: A-weighted energetic average level during the 23:00–07:00 night period.

*L*_AS,max,traffic_: A-weighted maximum level (slow [1 s] time filter) of traffic noise events.

For traffic noise exposure, we used existing high-fidelity audio recordings of aircraft, road and rail traffic from a previous study ^21^. There were 120 traffic noise events (40 each of road, rail and air), at five different maximum sound pressure levels (45–65 dB *L*_AS,max_ in 5 dB steps) across the night. We randomised the distribution of the length of intervals between noise events (3 to 5 minutes) and maximum sound pressure levels to obtain an even distribution throughout the night (23:00-07:00). For each traffic mode and maximum level, two specific sounds were used, based on having been identified as the most sleep-disturbing variants that induced the highest elevations in the Odds Ratio Product [ORP]^22^. The same traffic noise audio file was used at the same sound pressure levels for all nights with traffic noise across all subjects.

For the “pink noise*”* exposure, we used an audio file of continuous pink noise (20 to 20,000 Hz). This was played at a constant level of 45 dB *L*_AEq_ continuously during the night. This level corresponds to the lowest maximum value of the traffic noise, and is comparable ( + 1.4 dB) to the average night level of the traffic noise.

We performed daily checks to ensure that noise exposure was administered correctly during the preceding night. There were no instances of technical failure or incorrect noise playback in any study nights.

### Participants

Twelve healthy participants (mean age 23.6 years, range 21–29 years; 5 females, 7 males) were recruited in March and April 2022, via public advertisement around the University of Gothenburg campus and online. As a pilot, a sample size of 12 was expected to allow detection of physiological and self-reported effects of high-level environmental exposure^23^, and also allowed for balance in the randomised design. There were no interim analyses or stopping guidelines to inform early stopping or sample size re-estimation.

Participants were habitually good sleepers (PSQI < 5) with habitual mean bedtimes and rise times closely aligning with the experimental sleep opportunity times, determined with actigraphy in the week immediately prior to the study. They did not suffer from any sleep disorder, use any sleep medications or medications with potential side effects impacting sleep, or use sound machines or other similar machines or apps to aid their sleep. They were not at high risk for obstructive sleep apnoea, assessed via the STOP-BANG questionnaire^24^ and confirmed during the habituation night in the study (Apnoea Hypopnea Index <15). All participants had normal hearing, which was assessed via pure tone audiometry to 20 dB HL.

Two subjects dropped out after completing three nights in the study. Their data are included in the analysis, where available. One subject removed the PSG sensors during their sleep partway through one night; therefore, data from this night were not included in the analysis of PSG outcomes. No other data were missing.

Summary data for the 12 study subjects are given in Table 2. Half of the subjects were evening types, and half were morning types. All subjects had good habitual sleep quality. Physical and mental health were in the normal range (a mean ± SD physical health score 53.6 ± 7.36 is the norm for adults aged 18–34 years; a mean mental health score 48.9 ± 10.19 is the norm in the same age range)^25^. Most subjects rated their own bedroom as quiet. Annoyance and sleep disturbance at home were low for all noise sources. Sleep-wake times at home in the week immediately before the study closely aligned with the scheduled sleep-wake times in the laboratory protocol (23:00–07:00).

**Table 2:** Study subject characteristics

Variable	Level	*N* (%) or mean ± SD
Sex	Female	5 (41.7%)
	Male	7 (58.3%)
Age		23.6 ± 2.4 years
Chronotype	Extreme morning	1 (8.3%)
	Moderate morning	5 (41.7%)
	Intermediate	0 (0%)
	Moderate evening	5 (41.7%)
	Extreme evening	1 (8.3%)
Habitual sleep quality (PSQI)		2.2 ± 1.1
Noise sensitivity		65.8 ± 12.1
SF-36 Physical health		57.0 ± 4.4
SF-36 Mental Health		48.9 ± 10.2
Lights out time at home^a^		23:32 ± 01:23
Rise time at home^a^		07:47 ± 01:20
Perceived bedroom noise environment	Very quiet	2 (16.7%)
	Rather quiet	8 (66.7%)
	Not especially quiet	1 (8.3%)
	Rather noisy	1 (8.3%)
	Very noisy	0 (0%)
Annoyance, road noise	0–10 scale	1.6 ± 2.6
Annoyance, rail noise	0–10 scale	0.0 ± 0.0
Annoyance, tram noise	0–10 scale	1.4 ± 2.5
Annoyance, aircraft noise	0–10 scale	0.2 ± 0.6
Annoyance, neighbour noise	0–10 scale	2.4 ± 2.2
Sleep disturbance, road noise	0–10 scale	0.4 ± 0.9
Sleep disturbance, rail noise	0–10 scale	0.0 ± 0.0
Sleep disturbance, tram noise	0–10 scale	0.5 ± 1.0
Sleep disturbance, aircraft noise	0–10 scale	0.1 ± 0.3
Sleep disturbance, neighbour noise	0–10 scale	1.5 ± 2.0

^a^Measured at home with actigraphy during the week before the study.

To verify subject compliance with the self-regulated lights-out time (23:00), we manually scored actigraphy data from the in-lab study period. Across all subjects and study nights, the mean ± SD lights-out time was 22:55 ± 00:13. This indicates generally good adherence to the protocol, although there were two instances where a participant fell asleep rather early at 22:16 and 21:43. There was no evidence of daytime napping in the actigraphy records.

### Sleep registration

Each night, we recorded physiologic sleep with unattended polysomnography (PSG) using an ambulatory sleep monitor (SOMNOmedics SOMNOscreen Plus, Randersacker, Germany). Sensor attachments were performed in accordance with American Academy of Sleep Medicine guidelines^26^. Sleep scoring was done automatically using the Michele Sleep Scoring System (MSS) with manual verification^27^. We further analysed sleep using a novel marker of sleep depth and sleep disturbance. Specifically, the Odds Ratio Product (ORP)^28^, which is a continuous measure of sleep depth and stability determined in 3-second epochs. This approach provides measures of overall sleep architecture and the dynamics of changes in sleep across the night and in response to noise. ORP can reveal short-duration and/or subtle alterations in sleep activity, which nevertheless may be functionally relevant.

### Metabolomics

Each study morning, subjects provided a 2 ml blood sample for metabolomics analysis. Sample collection was done by a qualified clinician, and participants were passively observed for adverse effects (e.g., fainting) by the research team. No adverse events occurred during the study.

Blood samples were collected in Li-Hep plasma tubes (BD Vacutainer) and within 15 minutes centrifuged at 1300 × *g* for 10 minutes in accordance with manufacturer instructions. Plasma was aliquoted into cryovials (Sarstedt micro tube), then immediately transferred to -80 C freezers where they were stored until needed for analysis.

Plasma NMR samples were prepared by 50/50 buffer/sample dilution according to standard procedures. A Bruker IVDr platform was used to provide automated quantification of metabolites. Of the 40 metabolites investigated, 9 could not be analysed statistically because all concentrations were 0 or below the detection limit (ethanol, 2-aminobutyric acid, asparagine, creatine, 2-hydroxybutyric acid, citric acid, choline, 2-oxoglutaric acid, D-galactose).

Extreme and/or variable dietary behaviour can affect the metabolome/lipoprotein profile^29^. Therefore, each individual used a food diary to self-log all their meals, including snacks, throughout the laboratory study period. They were free to eat as they normally would, without constraints over the choice of particular foods, but were instructed that they must eat the same food for dinner/evening meal every day, and at the same time. The food diaries were returned to the study team at the end of the week and used to verify that the same meal was eaten daily. All food diaries indicated full compliance with the protocol, i.e., no within-subject variability in evening meal or snacks.

### Cognition

Chronic noise exposure can cause hyperactivity of the hypothalamic–pituitary–adrenal (HPA) axis in mice, leading to adverse cognitive effects, including reduced neural density and impairments in memory and motor coordination^30^. We tested whether nocturnal noise impacted cognitive performance using the Cognition test battery, fully described in Table S1 and Fig.  S1. Briefly, Cognition comprises of 10 computerised tests covering a range of cognitive domains: sensorimotor speed, spatial learning and memory, working memory, abstraction and concept formation, spatial orientation, emotion identification, abstract reasoning, complex scanning and visual tracking, risk decision making, and vigilant attention^31,32^. Cognition was administered every evening at 20:00 and every morning immediately after blood sampling. The administration during the first study evening was a training bout and was not included in the analysis. The mean ± SD duration to complete all 10 tests (excluding training bouts) was 18 m 29 s ± 3 m 10 s. Performance scores were checked against normative values after the first evening, the first morning, and every morning thereafter to identify any instances of misunderstanding the instructions or non-compliance. Any instances of misunderstanding instructions were always flagged and corrected after the first evening training bout. No indications of non-compliance were identified.

### Questionnaires

Baseline data were collected for noise sensitivity (Weinstein noise sensitivity scale^33^), habitual sleep quality (Pittsburgh Sleep Quality Index [PSQI]^34^), chronotype (single-question item), general physical and mental health (36-item Short Form Survey [SF-36]^35^), annoyance (ICBEN scale^36^) and sleep disturbance by different noise sources at home over the last 12 months, and perceived bedroom noise environment, and overall environmental sensitivity. These questionnaires were to accurately characterise the study participants and were not part of the selection criteria.

Each morning, subjects completed a questionnaire including different dimensions of sleep quality and disturbance in the preceding night^37^, sleepiness (Karolinska Sleepiness Scale [KSS])^38^, sleep disturbance by noise^36^, and sound-induced auditory fatigue^39^. Questionnaires were also completed each evening, including KSS, tiredness, tension and irritation. The evening questionnaires also contained items on relevant activities in the 8 h prior to bedtime, which could affect sleep: exercise, caffeine, nicotine, medications, and alcohol. Nicotine and alcohol were expressly prohibited during the study, and caffeine was prohibited after 15:00; therefore, these questions were used to check for subject compliance with the study protocol. Only KSS and auditory fatigue outcomes were prespecified in the clinical trial registry. We nevertheless include other questionnaire data, as they could capture relevant dimensions not captured by physiological measurements alone.

### Statistics and reproducibility

For each of the 10 *Cognition* tests, one key indicator of accuracy and one key indicator of speed were chosen as the main outcome variables of interest (see Smith et al.^40^ for full details). *Cognition* outcome data were corrected for practice and stimulus set difficulty effects prior to statistical analysis for a ≤ 5-day administration interval^41^. To facilitate comparisons between tests and the generation of summary scores, accuracy and speed outcomes of each test were z-transformed using the average and standard deviation of test performance at the start of the study period (excluding the first test bout, which was used for familiarising subjects with the *Cognition* battery). Speed scores were inverted during transformation so that higher z-scores always reflect better (i.e., faster, more accurate) performance. Summary accuracy and speed scores were calculated by averaging across cognitive domains. Risk taking on the BART was considered a separate category and not included in the accuracy summary score.

SF-36 data were used to calculate summary measures of physical health (PCS) and mental health (MCS) using the procedure of Ware^25^.

All statistical analyses were performed in SPSS version 29 (IBM Corp, Armonk, NY). Data were analysed in generalised linear mixed models (GLMMs) with study participants included as random effects (intercepts). This accounts for the similarity of repeated measurements made on the same individuals. Models were adjusted for sex and time in study. Model residuals were inspected visually to ensure conformity with model assumptions of normality. The habituation night was excluded from all analyses. For each outcome domain (physiological sleep, self-report, metabolomics, cognition), we used the false discovery rate (FDR) method to adjust for the number of statistical tests performed^42^. The critical value was <0.05.

We analysed two a priori selected measures of event-related change in ORP as dependent variables, following the approach of Smith et al.^22^. The first event-related outcome was the maximum change in ORP relative to the average ORP in the 30 s pre-noise baseline. The time window to calculate this event-related change was equal to the duration of each noise event. The second event-related outcome was the area under the ORP curve from the point of noise onset. Area under the curve (AUC) for event-related ORP change was calculated using the trapezoid rule in 3 s intervals corresponding to the 3 s width of ORP epochs. The average 3 s AUC during the 30 s pre-noise interval was subtracted from the AUC of each 3 s segment after noise onset, so that AUC data are individually normalised to pre-noise levels. Event-related AUC was calculated 80 s from noise onset, corresponding to the duration of the longest noise event.

In both event-related analyses, we implemented an adjusted GLMM that, in addition to the variables included in models of between-night effects (i.e., exposure condition, sex, and day in study), further included the following covariates: level of the noise event, traffic type (air, road or rail), sleep depth at noise onset (mean ORP in 30 s pre-noise period), ORP at noise onset, and time of night.

Sample sizes for all statistical analyses are given in Table 3.

**Table 3:** Sample sizes contributing to analyses for all outcomes

Outcome domain	Outcome variable	Totak sample size
Physiologic sleep	Event-related ORP change	*N* = 5280 events, from 43 person-nights within 12 subjects. Half (*N* = 2640) are sham events from Control and Pink noise only nights.
	Sleep macrostructure	*N* = 43 person-nights within 12 subjects
Questionnaires	Morning self-reported	*N* = 44 study days within 12 subjects
	Evening self-reported	*N* = 32 study days within 12 subjects^a^
Metabolomics	Blood metabolite concentrations	*N* = 44 study days within 12 subjects
Cognition	Morning test speed and accuracy outcomes	*N* = 44 study days within 12 subjects
	Evening test speed and accuracy outcomes	*N* = 32 study days within 12 subjects^a^

^a^Data for the evening after the final study night were not collected because participants had already exited the study.

## Results

### Changes in sleep depth and stability (ORP)

Here, we show that a traffic noise event induces event-related changes in sleep depth and stability. Averaged across all events, there is an increase in ORP following noise onset in the traffic noise night, peaking at 27–30 s before gradually decreasing towards pre-noise baseline ORP levels (Fig. 1B). Comparison with the quiet control condition confirms that these acute noise-induced shifts towards wakefulness and unstable sleep significantly exceed endogenous fluctuations in sleep, for both the peak ORP increase (*p* < 0.0001; Table S2) and the area under the ORP curve (*p* < 0.0001; Fig. 1C and Table S3). Elevations of ORP are stronger for higher noise levels (*p* < 0.0001; Fig. 1D and Tables S2 and S3), further confirming the sleep-fragmenting effect of noise.

When continuous pink noise was introduced throughout the night, the increase in ORP occurred more slowly (Fig. 1B), reaching significantly lower peak ORP values (*p* = 0.003; Table S2) and with reduced area under the ORP curve (*p* < 0.0001; Fig. 1C and Table S3). The level of pink noise (45 dB) corresponded to the maximum level of the quietest events (45 dB L_AS,max_), and accordingly, the sleep-disturbing effect of these lowest-level traffic events is fully attenuated (Fig. 1D). These data show that noise-induced sleep fragmentation is attenuated by the presence of a neutral auditory masker. Pink noise alone does not lead to any significant changes in sleep compared to the control condition.

Effects of noise exposure on measures of sleep macrostructure are given in Table S4. Exposure condition is significantly associated with the total number of EEG arousals (p < 0.00001) and the index of the hourly frequency of EEG arousals and awakenings (*p* < 0.00001). Post-hoc contrasts indicate an increased frequency of arousals and awakenings in both the Traffic noise only and the Pink + Traffic noise nights compared to both the Control and the Pink noise only night (all contrasts *p* < 0.01). There are no differences between the Control and Pink noise only conditions or between the Traffic noise only and the Pink + Traffic noise conditions. Associations between noise exposure and EEG alpha intrusions, minimum ORP during the first half of non-REM sleep, total sleep time, or percentage of sleep time in REM sleep do not survive FDR correction.

### Blood metabolome

**Fig. 2:**
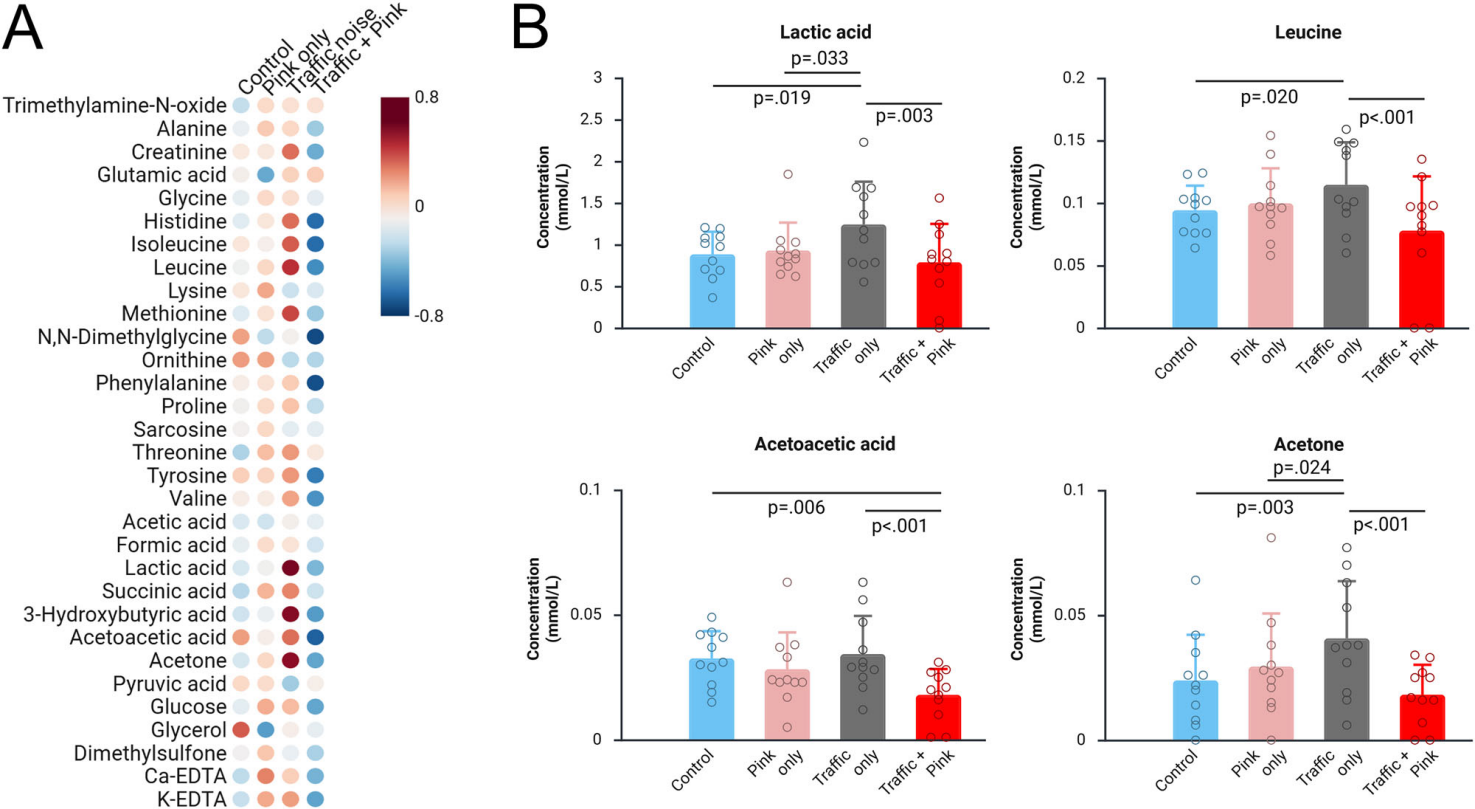
Effect of noise exposure on blood plasma metabolite concentrations measured each study morning. **A** Standardised (z-scored) metabolite concentrations. **B** Concentrations of metabolites that were significantly affected by noise exposure (columns and error bars indicate mean ± SD), adjusted for sex and time (days) in the study. *N* = 11 per exposure condition (total *N* = 44).

Standardised (z-scored) metabolite concentrations are given in Fig. 2A, see Table S5 for mean values and statistical test data. Effects on 3-hydroxybutyric acid, isoleucine, N,N-dimethylglycine and tyrosine did not survive FDR correction. After FDR correction, there are significant effects of exposure on lactic acid (*p* = 0.003), leucine (*p* = 0.005), acetoacetic acid (*p* = 0.005) and acetone (*p* = 0.004), see Fig. 2B. Within-subject time course data are given in Figs. S2–S5. Compared to the quiet control, after the traffic noise at night, there are significantly higher concentrations of lactic acid (*p* = 0.004), leucine (*p* = 0.020) and acetone (*p* = 0.003). When pink noise is introduced, these concentrations are reduced to around the control baseline levels (*p* > 0.1 for each contrast). The response pattern is different for acetoacetic acid, where concentrations are significantly lower after the night with traffic and pink noise compared to both the quiet control night (*p* = 0.006) and the traffic noise only night (*p* = 0.001).

To examine further the role of sleep disturbance in the relationship between noise exposure and metabolite concentrations, we post-hoc added the EEG arousal and awakening index to the GLMMs. Results are given in Tables S6 and S7. The arousal and awakening index is not directly associated with lactic acid, leucine, acetoacetic acid or acetone. Nor does it significantly change the overall model when added as a covariate to the analysis of associations between noise and these metabolites.

### Self-report

**Fig. 3:**
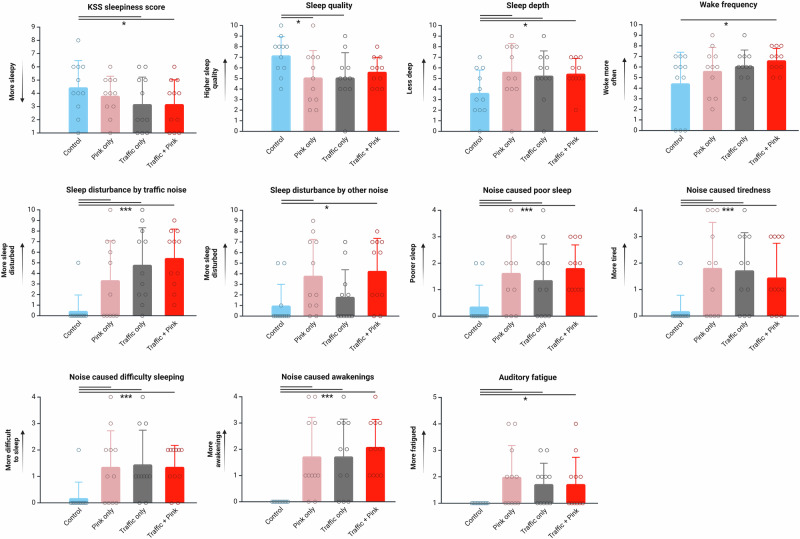
Self-report outcomes with a significant effect of exposure (columns and error bars indicate mean ± SD). Testing was performed in GLMMs (two-sided) with random subject effects. Asterisks denote adjusted pairwise comparisons *p < 0.05; ***p < 0.001 (see Table S8 for exact values). All ordinates are scaled to the full range of possible question response scales. For all questions *N* = 11 per exposure condition (total *N* = 44).

Results of the morning questionnaire data are given in Table S8. Eleven outcomes (58%) are significantly associated with noise exposure (Fig. 3). Subjective sleepiness is higher after nights with traffic noise and traffic plus pink noise. Sleep quality and sleep depth are perceived as worse after nights with pink noise or traffic noise. Sleep depth is also perceived as worse after the traffic + pink noise night, potentially because recalled awakenings are also higher during this night compared to the quiet control. During nights with any noise exposure at all (pink noise, traffic noise, or traffic + pink noise), noise is perceived as disturbing sleep, causing poor sleep, causing tiredness, causing difficulty sleeping, and causing awakenings. The presence of any noise exposure also causes some sound-induced auditory fatigue, although mean values are at or below the second lowest response choice on the scale.

There are no effects of exposure condition on evening questionnaire outcomes (Table S9).

### Cognition

There are no significant associations between exposure condition and cognitive speed or accuracy across any of the 10 individual tests in the morning (Table S10) or evening (Table S11) administrations. There are accordingly no significant effects on overall speed or overall accuracy, averaged across all cognitive domains (Fig.  S6).

## Discussion

Intermittent noise impacted sleep fragmentation and downstream changes in specific metabolite concentrations, whereas continuous noise (pink) at a similar level had no effect on these outcomes. This study shows that environmental noise at night directly induces metabolic changes that, in the long term, may contribute to the development of cardiometabolic disease.

Acute noise-induced disturbance of sleep was anticipated and has been shown in both laboratory and field studies^21,22,43^. That these acute effects translate to only minor or null effects in the overall structure of sleep across the night is also aligned with previous laboratory studies^21,22,44–46^. Noise-induced arousals can replace spontaneous arousals that occur endogenously during sleep^21^, nevertheless, evoking these reactions indicates that sleep continuity and stability are disturbed. The elevations in metabolite concentrations indicate these sleep disturbances are followed by metabolic changes, described further below, which are plausibly relevant for disease risk in the long term.

Results support the use of ORP as a measure of acute sleep disturbance. This is in alignment with our previous work, demonstrating that the ORP was more sensitive to the effects of noise on sleep than classical sleep scoring techniques^22^. ORP offers advantages over classical EEG arousal scoring, including more granular resolution of sleep depth and increased temporal precision^28^. By capturing subtle yet clinically-relevant effects of sleep disturbance, ORP is a biomarker that could offer mechanistic insights in public health and clinical research into not only noise, but potentially other environmental stressors, including vibration, artificial light and heat.

Lactate is a product of glycolysis and is reduced in the brain during sleep, reflecting decreased glycolysis as well as increased glymphatic clearance^47^. Blood levels of lactate are also lower during sleep^47^, and levels may be increasingly low for deeper stages of sleep^48^. Conversely, lactate is increased by wake-associated activities, including cortical arousal and muscle movements^47,49^, and during REM sleep^50^. Elevations in lactic acid after the traffic noise-only condition could therefore be a consequence of changes in brain metabolism following the increased cortical arousal and shifts towards wakefulness and unstable sleep after acute exposure to noise. Recent data show elevated levels of lactic acid in animals with depression-like symptoms induced by environmental stress, specifically blue light exposure, during sleep^51^. Lactic acid may therefore serve as a useful biomarker of changes in metabolic brain state in response to environmental stressors, including noise.

Acetone, which is one of the three endogenous ketone bodies, was elevated after the night of traffic noise. Elevated concentrations of the second endogenous ketone body, 3-hydroxybutyric acid, did not survive statistical correction for multiple testing. Concentrations of a third ketone body, acetoacetic acid, were lower after traffic + pink noise together. These three ketone bodies are produced by the liver and used peripherally for energy during limited glucose availability^52^. When glycogen reserves are low, there is a metabolic switch from using carbohydrates and glucose to fatty acids and ketones as cellular fuel^53^. Ketones can cross the blood-brain barrier and therefore serve as an alternative energy source during these periods. The changes in ketone concentrations, together with no significant changes in circulating glucose, could indicate alterations in metabolically-expensive brain energy consumption when exposed to noise during sleep^47,54^. However, the limited sample size means that potential effects on glucose levels may not have been detected. Further, effects on brain energy consumption remain speculative without markers of brain energy metabolism such as ATP^47,55^.

Alternatively, changes in insulin resistance due to noise-disturbed sleep may be responsible. Acute sleep disturbance can lead to decreased insulin levels^56^. Exposure to four nights of traffic noise has previously been found to impair glucose tolerance and insulin sensitivity, even while overall sleep macrostructure was preserved^12^. Insulin dysregulation would impair the utilisation of glucose, leading to elevated ketones. This hypothesis aligns with some epidemiological data, which has shown a higher risk for diabetes and diabetes mortality among people exposed to high levels of traffic noise^15,57^. Changes in insulin regulation could also explain the increased leucine levels. Leucine is an essential branched-chain amino acid that promotes protein synthesis and facilitates energy metabolism^58^. Our observed increase in circulating leucine indicates an impairment in the body’s ability to utilise or catabolise leucine. Increased leucine is associated with insulin resistance and decreased insulin secretion^59^. Impairments in leucine uptake could therefore contribute to insulin dysregulation.

Results are mixed regarding the efficacy of continuous pink noise as a sleep aid. On the one hand, pink noise attenuated the acute sleep-fragmenting and metabolic effects of traffic noise. This is in line with previous work showing that when raising the background level in this way, the number of EEG arousals induced by intensive care unit (ICU) noise is reduced^20^. The reduction in ORP elevations across all maximum sound pressure levels indicates that the difference between background and maximum levels of noise events is of more relevance for acute response than the maximum noise levels per se. Pink noise may therefore be helpful to mitigate against physiological sleep disturbance in environments characterised by high-level noise events, including hospitals (especially ICUs), residences close to major traffic routes, and certain occupational settings such as ship or truck cabins.

On the other hand, pink noise led to no significant improvements in sleep macrostructure compared to the quiet condition. Further, pink noise provided no benefit to any measures of subjective sleep or restoration, and in fact, sound-induced auditory fatigue and several measures of perceived sleep quality and sleep disturbance were negatively affected relative to the quiet condition. In a small (*n* = 6) experimental study, Terzano et al. found a positive linear relationship between increasing broadband sound from 45 dB to 75 dB and increased wakefulness, reduced total sleep time, reduced slow wave sleep and more fragmented sleep^60^. Observational epidemiological studies show that higher environmental noise levels across the night are positively associated with self-reported sleep disturbance^61^. Levels of pink noise lower than 45 dB would thus presumably be perceived as less directly disturbing, yet would less effectively mask disturbing noise events. Conversely, higher levels of broadband noise throughout the night would more effectively mask traffic noise, but may also actively disrupt sleep.

The lack of an extended quiet period during noise nights, because of either the continuous pink noise or the short time between traffic noise events, may have precluded sufficient rest for the auditory system. The use of white noise therapy in the treatment of tinnitus may lead to changes in functional and structural integrity of the central auditory system and the brain^62^. The short-term use of sound machines may therefore offer benefits against the disturbing effects of noise, but their long-term use should be considered carefully.

Noise exposure was not associated with significant changes in cognitive performance. A past study using the same cognitive test battery found that accumulating sleep debt due to partial sleep restriction over six weeks leads to decreases in overall accuracy across cognitive domains in high-performing adults^40^. However, sleep disturbance by noise is probably less cognitively impactful than chronic sleep restriction. Thus, we anticipate smaller effect sizes that we would be underpowered to detect in the current pilot study. Future studies should include larger sample sizes, and also consider using longer test durations, which might be more sensitive to the cognitive effects of sleep loss^63^.

A limitation of this study is the small sample size and homogeneity of the study group. Effects of noise on sleep and the metabolome may differ in the more general population, due to age-related changes in sleep quality^64^, noise exposure history and possible habituation (including regular sound machine use)^65^, higher prevalence of sleep disorders^66^, and interindividual variability^67^. Thus, the current data may not be fully generalisable, particularly among vulnerable or sensitive groups. The sample size also means that positive or negative effects of pink noise, beyond those observed, may not have been detected. A further limitation is that only short-term effects were captured. This could explain the lack of effects on cognitive outcomes, as cognitive effects of moderate sleep disturbance may manifest only over longer time scales^40^. Further, longitudinal and cohort studies are required to confirm if the acute changes in metabolite concentrations we observed translate to long-term metabolic changes and elevated disease risk.

Because of the purposeful decision to minimise the number of sensors in the PSG montage, we could not perform a full clinical assessment to confirm the self-reported absence of all sleep disorders. The reliance on subjects self-enforcing the lights-out time, combined with limitations of actigraphy in accurately detecting lights-out time, may have resulted in some misclassification of the true lights-out time. However, the only sleep data this would affect are the total wake time and sleep efficiency, neither of which was affected by exposure condition. Reliance on self-report is also a limitation for the food diaries, as non-compliance with the requirement to always eat the same evening meal may go unreported. The concentrations of next-morning fasting metabolites will be driven primarily by dinner timing and composition^68^, but we cannot completely exclude small and non-systematic effects of ad-lib food intake prior to the evening meal.

As an exploratory pilot study, we did not include some biological measurements that would have offered additional insights into the metabolic mechanisms affected. In particular, insulin levels could be used to determine the homeostatic model assessment for insulin resistance and beta-cell function^69^. Future research incorporating such measurements will be valuable in further revealing the metabolic pathways linking noise and disease.

A major strength of this work is the high control over nighttime exposure to noise, vibration, ambient light, and dietary intake afforded by the laboratory environment. These stressors can influence sleep, metabolism and their interaction. The laboratory environment itself was set up to maximise ecological validity, and we have previously shown that noise-induced sleep disturbance in this setting is comparable with data obtained in the field^70^. Participants were free to follow their normal daytime routine, and thus, results were not obtained during artificially restrictive situations. As such, we expect the results to be representative of typical situations occurring among people exposed to environmental noise at home.

## Conclusion

Nights with acutely disturbing noise are accompanied by subjective sleep disturbance and changes in metabolite concentrations that suggest changes in metabolic processes in response to noise-induced cortical arousal. Attenuating these arousals with a pink noise masker accordingly attenuates the markers of metabolic function, but subjective outcomes are not significantly improved. These data provide mechanistic insights that help explain epidemiological relationships between environmental stressors during sleep and chronic disease^2^. Results also highlight the importance of considering metrics of noise exposure that capture more than a single average noise level, and physiological effects beyond the questionnaire-derived exposure-response functions, that are traditionally used to inform public health noise guidelines and policy^71^.

## Supplementary information

Supplementary Information

Description of Additional Supplementary Files

Supplementary Data 1

## Data Availability

The datasets generated during the current study are available from the authors on reasonable request (the responsible person for replying to requests is the corresponding author). All source data underlying the main figures are available as Supplemental Data accompanying this paper.

## References

[1] Aasvang, G. M. et al. Burden of disease due to transportation noise in the Nordic countries. *Environ. Res.* **231**, 116077 (2023). 37156356 10.1016/j.envres.2023.116077

[2] van Kempen, E., Casas, M., Pershagen, G. & Foraster, M. WHO environmental noise guidelines for the European region: a systematic review on environmental noise and cardiovascular and metabolic effects: a summary. *Int. J. Environ. Res. Public Health* **15**, 379 (2018). 10.3390/ijerph15020379 PMC5858448 29470452

[3] van Kamp, I., Simon, S., Notley, H., Baliatsas, C. & van Kempen, E. Evidence relating to environmental noise exposure and annoyance, sleep disturbance, cardio-vascular and metabolic health outcomes in the context of IGCB (N): a scoping review of new evidence. *Int J. Environ. Res. Pub. Health* **17**, 3016 (2020). 32357581 10.3390/ijerph17093016 PMC7246943

[4] Sørensen, M. et al. Long-term exposure to transportation noise and risk of type 2 diabetes: a cohort study. *Environ. Res.* **217**, 114795 (2023). 36402187 10.1016/j.envres.2022.114795

[5] Saucy, A. et al. Does night-time aircraft noise trigger mortality? A case-crossover study on 24 886 cardiovascular deaths. *Eur. Heart J.* **42**, 835–843 (2020). 10.1093/eurheartj/ehaa957 PMC7897463 33245107

[6] Gottlieb, D. J. et al. Association of usual sleep duration with hypertension: the Sleep Heart Health Study. *Sleep* **29**, 1009–1014 (2006). 16944668 10.1093/sleep/29.8.1009

[7] Taheri, S., Lin, L., Austin, D., Young, T. & Mignot, E. Short sleep duration is associated with reduced leptin, elevated ghrelin, and increased body mass index. *PLoS Med.* **1**, e62 (2004). 15602591 10.1371/journal.pmed.0010062 PMC535701

[8] Münzel, T., Gori, T., Babisch, W. & Basner, M. Cardiovascular effects of environmental noise exposure. *Eur. Heart J.* **35**, 829–836 (2014). 24616334 10.1093/eurheartj/ehu030 PMC3971384

[9] Herzog, J. et al. Acute exposure to nocturnal train noise induces endothelial dysfunction and pro-thromboinflammatory changes of the plasma proteome in healthy subjects. *Basic Res. Cardiol.* **114**, 43 (2019). 10.1007/s00395-019-0753-y PMC6817813 31664594

[10] Schmidt, F. P. et al. Effect of nighttime aircraft noise exposure on endothelial function and stress hormone release in healthy adults. *Eur. Heart J.* **34**, 3508–3514a (2013). 23821397 10.1093/eurheartj/eht269 PMC3844151

[11] Münzel, T. et al. Adverse cardiovascular effects of traffic noise with a focus on nighttime noise and the new WHO noise guidelines. *Annu. Rev. Public Health* **41**, 309–328 (2020). 31922930 10.1146/annurev-publhealth-081519-062400

[12] Thiesse, L. et al. Adverse impact of nocturnal transportation noise on glucose regulation in healthy young adults: Effect of different noise scenarios. *Environ. Int.* **121**, 1011–1023 (2018). 30408889 10.1016/j.envint.2018.05.036

[13] Buxton, O. M. et al. Sleep restriction for 1 week reduces insulin sensitivity in healthy men. *Diabetes* **59**, 2126–2133 (2010). 20585000 10.2337/db09-0699 PMC2927933

[14] Spiegel, K., Tasali, E., Leproult, R. & Van Cauter, E. Effects of poor and short sleep on glucose metabolism and obesity risk. *Nat. Rev. Endocrinol.* **5**, 253–261 (2009). 19444258 10.1038/nrendo.2009.23 PMC4457292

[15] Zare Sakhvidi, M. J., Zare Sakhvidi, F., Mehrparvar, A. H., Foraster, M. & Dadvand, P. Association between noise exposure and diabetes: a systematic review and meta-analysis. *Environ. Res.* **166**, 647–657 (2018). 30006240 10.1016/j.envres.2018.05.011

[16] Liu, X. & Locasale, J. W. Metabolomics: a primer. *Trends Biochem. Sci.* **42**, 274–284 (2017). 28196646 10.1016/j.tibs.2017.01.004 PMC5376220

[17] Malik, D. M., Paschos, G. K., Sehgal, A. & Weljie, A. M. Circadian and sleep metabolomics across species. *J. Mol. Biol.* **432**, 3578–3610 (2020). 32376454 10.1016/j.jmb.2020.04.027 PMC7781158

[18] McGarrah, R. W., Crown, S. B., Zhang, G. F., Shah, S. H. & Newgard, C. B. Cardiovascular metabolomics. *Circ. Res.* **122**, 1238–1258 (2018). 29700070 10.1161/CIRCRESAHA.117.311002 PMC6029726

[19] Riedy, S. M., Smith, M. G., Rocha, S. & Basner, M. Noise as a sleep aid: a systematic review. *Sleep. Med. Rev.* **55**, 101385 (2021). 33007706 10.1016/j.smrv.2020.101385

[20] Stanchina, M. L., Abu-Hijleh, M., Chaudhry, B. K., Carlisle, C. C. & Millman, R. P. The influence of white noise on sleep in subjects exposed to ICU noise. *Sleep. Med.* **6**, 423–428 (2005). 16139772 10.1016/j.sleep.2004.12.004

[21] Basner, M., Müller, U. & Elmenhorst, E. M. Single and combined effects of air, road, and rail traffic noise on sleep and recuperation. *Sleep* **34**, 11–23 (2011). 21203365 10.1093/sleep/34.1.11 PMC3001788

[22] Smith, M. G. et al. Traffic noise-induced changes in wake-propensity measured with the Odds-Ratio Product (ORP). *Sci. Total Environ.* **805**, 150205 (2022). 10.1016/j.scitotenv.2021.150191 34818802

[23] Smith, M. G., Croy, I., Ogren, M. & Persson Waye, K. On the influence of freight trains on humans: a laboratory investigation of the impact of nocturnal low frequency vibration and noise on sleep and heart rate. *PloS One* **8**, e55829 (2013). 23409055 10.1371/journal.pone.0055829 PMC3567002

[24] Chung, F. et al. STOP questionnaire: a tool to screen patients for obstructive sleep apnea. *Anesthesiol. (Phila.)* **108**, 812–821 (2008). 10.1097/ALN.0b013e31816d83e4 18431116

[25] Ware, J. E., Jr., Kosinski, M. & Keller, S. D. *SF-36 Physical and Mental Health Summary Scales: A User's Manual*. (Health Assessment Lab, New England Medical Center, 1994).

[26] Iber, C., Ancoli-Israel, S., Chesson, A. L. & Quan, S. F. *The AASM Manual for the Scoring of Sleep and Associated Events: Rules, Terminology and Technical Specifications*. 1st edn, 1–59 (American Academy of Sleep Medicine, 2007).

[27] Malhotra, A. et al. Performance of an automated polysomnography scoring system versus computer-assisted manual scoring. *Sleep* **36**, 573–582 (2013). 23565003 10.5665/sleep.2548 PMC3612255

[28] Younes, M. et al. Odds ratio product of sleep EEG as a continuous measure of sleep state. *Sleep* **38**, 641–654 (2015). 25348125 10.5665/sleep.4588 PMC4355904

[29] Guasch-Ferré, M., Bhupathiraju, S. N. & Hu, F. B. Use of metabolomics in improving assessment of dietary intake. *Clin. Chem.* **64**, 82–98 (2018). 29038146 10.1373/clinchem.2017.272344 PMC5975233

[30] Jafari, Z., Kolb, B. E. & Mohajerani, M. H. Chronic traffic noise stress accelerates brain impairment and cognitive decline in mice. *Exp. Neurol.* **308**, 1–12 (2018). 29936225 10.1016/j.expneurol.2018.06.011

[31] Basner, M. et al. Development and validation of the cognition test battery for spaceflight. *Aerosp. Med. Hum. Perform.* **86**, 942–952 (2015). 26564759 10.3357/AMHP.4343.2015 PMC4691281

[32] Nasrini, J. et al. Cognitive performance during confinement and sleep restriction in NASA's human exploration research analog (HERA). *Front. Physiol.* **11**, 394 (2020). 32411017 10.3389/fphys.2020.00394 PMC7198903

[33] Weinstein, N. D. Individual differences in reactions to noise: a longitudinal study in a college dormitory. *J. Appl Psychol.* **63**, 458–466 (1978). 701213

[34] Buysse, D. J., Reynolds, C. F. 3rd, Monk, T. H., Berman, S. R. & Kupfer, D. J. The Pittsburgh Sleep Quality Index: a new instrument for psychiatric practice and research. *Psychiatry Res.* **28**, 193–213 (1989). 2748771 10.1016/0165-1781(89)90047-4

[35] Ware, J. E. Jr. & Sherbourne, C. D. The MOS 36-item short-form health survey (SF-36). I. Conceptual framework and item selection. *Med Care* **30**, 473–483 (1992). 1593914

[36] Fields, J. M. et al. Standardized general-purpose noise reaction questions for community noise surveys: Research and a recommendation. *J. Sound Vib.* **242**, 641–679 (2001).

[37] Croy, I., Smith, M. G., Gidlöf-Gunnarsson, A. & Persson Waye, K. Optimal questions for sleep in epidemiological studies: Comparisons of subjective and objective measures in laboratory and field studies. *Behav. Sleep. Med.* **15**, 466–482 (2017). 27159152 10.1080/15402002.2016.1163700

[38] Åkerstedt, T. & Gillberg, M. Subjective and objective sleepiness in the active individual. *Int. J. Neurosci.* **52**, 29–37 (1990). 2265922 10.3109/00207459008994241

[39] Fredriksson, S., Hammar, O., Magnusson, L., Kahari, K. & Persson Waye, K. Validating self-reporting of hearing-related symptoms against pure-tone audiometry, otoacoustic emission, and speech audiometry. *Int. J. Audio.* **55**, 454–462 (2016). 10.1080/14992027.2016.1177210 27195802

[40] Smith, M. G. et al. Effects of six weeks of chronic sleep restriction with weekend recovery on cognitive performance and wellbeing in high-performing adults. *Sleep* **44**, zsab051 (2021). 10.1093/sleep/zsab051 33630069

[41] Basner, M. et al. Cognition test battery: adjusting for practice and stimulus set effects for varying administration intervals in high performing individuals. *J. Clin. Exp. Neuropsychol.* **42**, 444–457 (2020). 10.1080/13803395.2020.1773765 PMC7375457 32539487

[42] Benjamini, Y. & Hochberg, Y. Controlling the false discovery rate - a practical and powerful approach to multiple testing. *J. R. Stat. Soc. B* **57**, 289–300 (1995).

[43] Basner, M. & McGuire, S. WHO Environmental Noise Guidelines for the European Region: A Systematic Review on Environmental Noise and Effects on Sleep. *Int. J. Environ. Res. Public Health* **15**, 519 (2018). 10.3390/ijerph15030519 PMC5877064 29538344

[44] Smith, M. G., Ogren, M., Ageborg Morsing, J. & Persson Waye, K. Effects of ground-borne noise from railway tunnels on sleep: a polysomnographic study. *Build Environ.* **149**, 288–296 (2019).

[45] Smith, M. G. et al. Physiological effects of railway vibration and noise on sleep. *J. Acoust. Soc. Am.* **141**, 3262–3269 (2017). 28599531 10.1121/1.4983302 PMC5433882

[46] Rudzik, F. et al. Sleep spindle characteristics and arousability from nighttime transportation noise exposure in healthy young and older individuals. *Sleep* **41**, zsy077 (2018). 10.1093/sleep/zsy077 29697833

[47] Aalling, N. N., Nedergaard, M. & DiNuzzo, M. Cerebral metabolic changes during sleep. *Curr. Neurol. Neurosci. Rep.* **18**, 57 (2018). 30014344 10.1007/s11910-018-0868-9 PMC6688614

[48] Yildiz, S. et al. Sleep dependent changes of lactate concentration in human brain. *bioRxiv*, 2021.2012.2005.471196 (2021).

[49] Zuend, M. et al. Arousal-induced cortical activity triggers lactate release from astrocytes. *Nat. Metab.* **2**, 179–191 (2020). 32694692 10.1038/s42255-020-0170-4

[50] Naylor, E. et al. Lactate as a biomarker for sleep. *Sleep* **35**, 1209–1222 (2012). 22942499 10.5665/sleep.2072 PMC3413798

[51] Li, Y. et al. Lactic acid contributes to the emergence of depression-like behaviors triggered by blue light exposure during sleep. *Ecotoxicol. Environ. Saf.* **289**, 117643 (2025). 39756180 10.1016/j.ecoenv.2024.117643

[52] Laffel, L. Ketone bodies: a review of physiology, pathophysiology and application of monitoring to diabetes. *Diab. Metab. Res. Rev.* **15**, 412–426 (1999). 10.1002/(sici)1520-7560(199911/12)15:6<412::aid-dmrr72>3.0.co;2-8 10634967

[53] Mattson, M. P., Moehl, K., Ghena, N., Schmaedick, M. & Cheng, A. Intermittent metabolic switching, neuroplasticity and brain health. *Nat. Rev. Neurosci.* **19**, 63–80 (2018). 29321682 10.1038/nrn.2017.156 PMC5913738

[54] Padamsey, Z. & Rochefort, N. L. Paying the brain's energy bill. *Curr. Opin. Neurobiol.* **78**, 102668 (2023). 36571958 10.1016/j.conb.2022.102668

[55] Cunnane, S. C. et al. Brain energy rescue: an emerging therapeutic concept for neurodegenerative disorders of ageing. *Nat. Rev. Drug Discov.* **19**, 609–633 (2020). 32709961 10.1038/s41573-020-0072-x PMC7948516

[56] Spiegel, K., Knutson, K., Leproult, R., Tasali, E. & Van Cauter, E. Sleep loss: a novel risk factor for insulin resistance and Type 2 diabetes. *J. Appl Physiol.* **99**, 2008–2019 (2005). 16227462 10.1152/japplphysiol.00660.2005

[57] Vienneau, D. et al. Long-term exposure to transportation noise and diabetes mellitus mortality: a national cohort study and updated meta-analysis. *Environ. Health* **23**, 46 (2024). 38702725 10.1186/s12940-024-01084-0 PMC11068573

[58] Duan, Y. et al. The role of leucine and its metabolites in protein and energy metabolism. *Amino Acids* **48**, 41–51 (2016). 26255285 10.1007/s00726-015-2067-1

[59] Vangipurapu, J., Stancáková, A., Smith, U., Kuusisto, J. & Laakso, M. Nine amino acids are associated with decreased insulin secretion and elevated glucose levels in a 7.4-year follow-up study of 5,181 Finnish men. *Diabetes* **68**, 1353–1358 (2019). 30885989 10.2337/db18-1076

[60] Terzano, M. G., Parrino, L., Fioriti, G., Orofiamma, B. & Depoortere, H. Modifications of sleep structure induced by increasing levels of acoustic perturbation in normal subjects. *Electroencephalogr. Clin. Neurophysiol.* **76**, 29–38 (1990). 1694482 10.1016/0013-4694(90)90055-o

[61] Smith, M. G., Cordoza, M. & Basner, M. Environmental noise and effects on sleep: an update to the WHO systematic review and meta-analysis. *Environ. Health Perspect.* **130**, 076001 (2022). 35857401 10.1289/EHP10197 PMC9272916

[62] Attarha, M., Bigelow, J. & Merzenich, M. M. Unintended consequences of white noise therapy for tinnitus-otolaryngology's Cobra effect: a review. *JAMA Otolaryngol. Head. Neck Surg.* **144**, 938–943 (2018). 30178067 10.1001/jamaoto.2018.1856

[63] Antler, C. A., Yamazaki, E. M., Casale, C. E., Brieva, T. E. & Goel, N. The 3-minute psychomotor vigilance test demonstrates inadequate convergent validity relative to the 10-minute psychomotor vigilance test across sleep loss and recovery. *Front. Neurosci.* **16**, 815697 (2022). 10.3389/fnins.2022.815697 PMC8885985 35242006

[64] Ohayon, M. M., Carskadon, M. A., Guilleminault, C. & Vitiello, M. V. Meta-analysis of quantitative sleep parameters from childhood to old age in healthy individuals: developing normative sleep values across the human lifespan. *Sleep* **27**, 1255–1273 (2004). 15586779 10.1093/sleep/27.7.1255

[65] Babisch, W. Cardiovascular effects of noise. *Noise Health* **13**, 201–204 (2011). 21537102 10.4103/1463-1741.80148

[66] Crowley, K. Sleep and sleep disorders in older adults. *Neuropsychol. Rev.* **21**, 41–53 (2011). 21225347 10.1007/s11065-010-9154-6

[67] McGuire, S., Müller, U., Elmenhorst, E. M. & Basner, M. Inter-individual differences in the effects of aircraft noise on sleep fragmentation. *Sleep* **39**, 1107–1110 (2016). 26856901 10.5665/sleep.5764 PMC4835309

[68] Beccuti, G. et al. Timing of food intake: sounding the alarm about metabolic impairments? A systematic review. *Pharmacol. Res.* **125**, 132–141 (2017). 28928073 10.1016/j.phrs.2017.09.005

[69] Matthews, D. R. et al. Homeostasis model assessment: insulin resistance and β-cell function from fasting plasma glucose and insulin concentrations in man. *Diabetologia* **28**, 412–419 (1985). 3899825 10.1007/BF00280883

[70] Persson Waye, K. et al. Assessing the exposure-response relationship of sleep disturbance and vibration in field and laboratory settings. *Environ. Pollut.* **245**, 558–567 (2019). 30469126 10.1016/j.envpol.2018.09.082

[71] World Health Organization. *Environmental Noise Guidelines for the European Region*. (World Health Organization Regional Office for Europe, 2018).

