# Supplementary material for: Pink noise reduces impact of traffic noise on sleep and the blood metabolome: a cross-over pilot study
Source: Commun Med (Lond). 2026 Jan 10;6:114. doi: 10.1038/s43856-026-01380-5 (PMC12901012; doi:10.1038/s43856-026-01380-5)
Supplement: Supplementary file 1 — Supplementary Information [file 43856_2026_1380_MOESM1_ESM.pdf]

Pink noise mitigates some of the negative effects of traffic noise on sleep and blood metabolome: Supplemental material

Natalia Vincens[1]; Anna Nause[1]; Mathias Basner[2]; Sofie Fredriksson[1,3]; Daniel Malmodin[4,5]; Anders Bay Nord[5]; Kerstin Persson Waye[1]; Magdy Younes[6]; Ding Zou[7]; Michael G. Smith[1]*

[1] School of Public Health and Community Medicine, Institute of Medicine, Sahlgrenska Academy, University of Gothenburg, Gothenburg, Sweden

[2] Unit for Experimental Psychiatry, Division of Sleep and Chronobiology, Department of Psychiatry, University of Pennsylvania Perelman School of Medicine, Philadelphia PA, USA

[3] Region Västra Götaland, Habilitation and Health, Hearing Organization, Gothenburg, Sweden

[4] National Bioinformatics Infrastructure Sweden (NBIS)

[5] Swedish NMR Centre, University of Gothenburg, Gothenburg, Sweden

[6] Sleep Disorders Centre, University of Manitoba, Winnipeg MB, Canada

[7] Center for Sleep and Vigilance Disorders, Sahlgrenska Academy, University of Gothenburg, Gothenburg, Sweden

*Corresponding author Michael.smith@amm.gu.se

Table S1 Overview of the *Cognition* test battery.

Test	Procedure	Cognitive domains assessed	Brain regions primarily recruited	Mean ± SD test duration (excluding training bout)
Motor Praxis (MP)	Click on squares that appear randomly on the screen, each successive square smaller and thus more difficult to track.	Sensorimotor speed	Sensorimotor cortex	40s ± 64s
Visual Object Learning (VOLT)	Memorize 10 sequentially displayed three-dimensional figures. Later, subjects are instructed to select those objects memorized from a set of 20 such objects also sequentially presented, some of them from the learning set and some of them new.	Spatial learning and memory	Medial temporal cortex, hippocampus	116s ± 24s
Fractal 2-Back (F2B)	Presentation of a set of fractals, each potentially repeated multiple times. Respond when the current stimulus matches the stimulus displayed two fractals ago	Working memory	Dorsolateral prefrontal cortex, cingulate, hippocampus	128s ± 26s
Abstract Matching (AM)	Subjects presented with two pairs of objects at the bottom left and right of the screen, varied on perceptual dimensions (e.g., color and shape). Subjects presented with a target object in the upper middle of the screen that they must classify as more belonging with one of the two pairs, based on a set of implicit, abstract rules.	Abstraction, concept formation	Prefrontal cortex	112s ± 28s
Line Orientation (LOT)	Presented with two lines at a time, one stationary and the other can be rotated. Subjects rotate the movable line until it is parallel to the stationary line.	Spatial orientation	Right temporo-parietal cortex, visual cortex	105s ± 33s
Emotion Recognition (ERT)	Presented with photographs of faces portraying emotional facial expressions of varying intensities. Subjects given a set of emotion labels (“happy”; “sad”; “angry”; “fearful”; and “no emotion”) and must select the label correctly describing the expressed emotion.	Emotion identification	Cingulate, amygdala, hippocampus, fusiform face area	71s ± 30s
Matrix Reasoning (MRT)	A series of patterns are overlaid on a grid. One element from the grid is missing, the subject must select the element that fits the pattern from a set of alternative options.	Abstract reasoning	Prefrontal cortex, parietal cortex, temporal cortex	126s ± 47s
Digit Symbol Substitution (DSST)	Subjects required to refer to a displayed legend relating each of the digits one through nine to specific symbols. One of the nine symbols appears on the screen and the subject selects the corresponding number as quickly as possible.	Complex scanning and visual tracking	Temporal cortex, prefrontal cortex, motor cortex	102s ± 4s
Balloon Analog Risk (BART)	Inflate an animated balloon or collect a reward. Participants are rewarded in proportion to the final size of each balloon, but a balloon will pop after a hidden number of pumps, which changes from trial to trial	Risk decision making	Orbital frontal and ventromedial prefrontal cortex, amygdala, hippocampus, anterior cingulate cortex, ventral striatum	129s ± 25s
Psychomotor Vigilance (PVT)	Monitor a box on the screen, and hit the space bar once a millisecond counter appears in the box and starts incrementing.	Vigilant attention	Prefrontal cortex, motor cortex, inferior parietal and some visual cortex	180s ± 1s

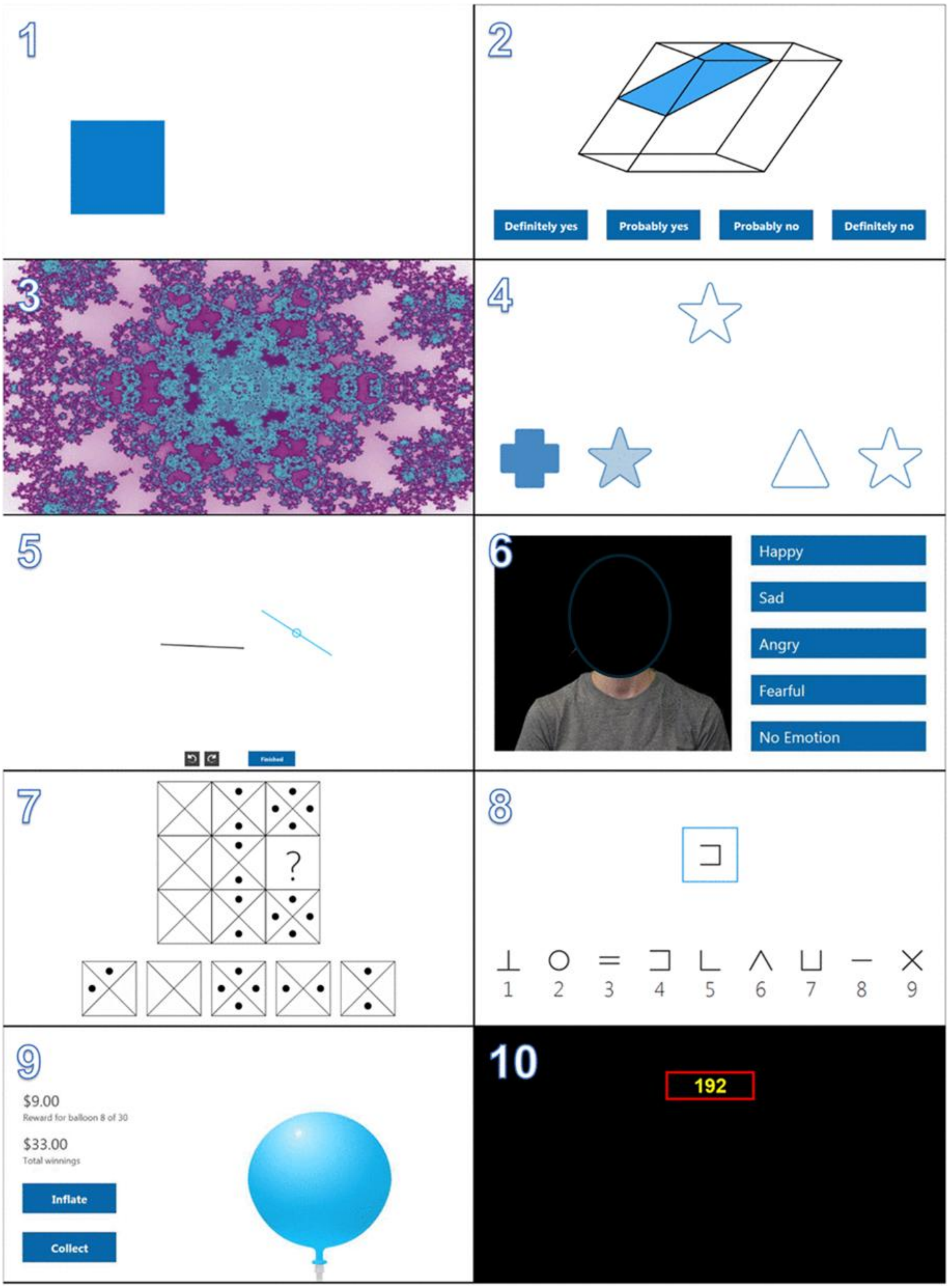

Figure S1 Screenshots of the 10 individual tests comprising the *Cognition* test battery. The tests are listed in the standard order of administration: 1) Motor Praxis (MP); 2) Visual Object Learning (VOLT); 3) Fractal 2-Back (F2B); 4) Abstract Matching (AM); 5) Line Orientation (LOT); 6) Emotion Recognition (ERT); 7) Matrix Reasoning (MRT); 8) Digit Symbol Substitution (DSST); 9) Balloon Analog Risk (BART); and 10) Psychomotor Vigilance (PVT). Note: the face in the ERT photograph has not been included in this publication.

Table S2 Results of regression model for maximum ORP change relative to 30s pre-noise baseline.

Variable	Type III Fixed effect		Level	EMM (95% CI) [a]	Post-hoc pairwise comparison p-values [b] Sub-headings indicate contrast level			
	Test statistic (df)	p-value						
					**Traffic noise**	**Pink noise**	**Traffic + Pink noise**	
Exposure condition	F(3, 5050) = 12.7	< .0001	Control	1.06 (1.02; 1.10)	< .0001	.618	.124	
			Traffic noise	1.17 (1.13; 1.21)	-	< .0001	.003	
			Pink noise	1.05 (1.01; 1.09)	-	-	.056	
			Traffic + Pink noise	1.10 (1.06; 1.14)	-	-	-	
					**50**	**55**	**60**	**65**
$L_{AS,max}$	F(4, 5050) = 48.0	< .0001	45	0.92 (0.88; 0.96)	< .0001	< .0001	< .0001	< .0001
			50	1.08 (1.04; 1.12)	-	.602	< .0001	< .0001
			55	1.10 (1.06; 1.14)	-	-	< .0001	.001
			60	1.20 (1.16; 1.25)	-	-	-	.602
			65	1.18 (1.14; 1.22)	-	-	-	-
					**Road**	**Rail**		
Traffic mode	F(2, 5050) = 216.3	< .0001	Air	1.30 (1.27; 1.34)	< .0001	< .0001		
			Road	0.95 (0.92; 0.99)	-	< .0001		
			Rail	1.03 (1.00; 1.07)	-	-		
Sex	F(1, 5050) = 2.6	.109	Male					
			Female					
				**Unstandardised regression coefficient (95% CI)**				
Baseline 30s ORP	F(1, 5050) = 1199.6	< .0001	-	0.017 (-0.631; -0.563)				
ORP at noise onset	F(1, 5050) = 352.9	< .0001	-	0.012 (0.198; 0.244)				
Time of night	F(1, 5050) = 32.8	< .0001	-	0.077 (0.288; 0.588)				
Night in study	F(1, 5050) = 0.8	.359	-	0.007 (-0.019; 0.007)				

EMM Estimated Marginal Means; CI Confidence Interval; df degrees of freedom.

[a] Covariates evaluated in the model at the following values: baseline 30s ORP=0.750; ORP at noise onset = 0.744; Time of night = 02:59; Night in study = 3.4

[b] Multiple testing corrections applied

Table S3 Results of regression model for area under the ORP curve.

Variable	Type III Fixed effect: Test statistic (df)	Type III Fixed effect: p-value	Level	EMM (95% CI) [a]	Post-hoc pairwise comparison p-values [b] (Sub-headings indicate contrast level)			
					**Traffic noise**	**Pink noise**	**Traffic + Pink noise**	
Exposure condition	F(3, 4942) = 28.4	< .0001	Control	-2.18 (-4.62; 0.25)	< .0001	.061	< .0001	
			Traffic noise	7.01 (4.55; 9.47)	-	< .0010	< .0001	
			Pink noise	-0.03 (-2.47; 2.4)	-	-	.068	
			Traffic + Pink noise	1.83 (-0.61; 4.27)	-	-	-	
					**50**	**55**	**60**	**65**
$L_{AS,max}$	F(4, 4942) = 13.2	< .0001	45	-0.96 (-3.5; 1.57)	.787	.189	.242	< .0001
			50	0 (-2.53; 2.53)	-	.780	.787	< .0001
			55	1.45 (-1.08; 3.97)	-	-	.865	< .0001
			60	1.26 (-1.28; 3.79)	-	-	-	< .0001
			65	6.54 (4; 9.08)	-	-	-	-
					**Road**	**Rail**		
Traffic mode	F(2, 4942) = 2.9	.058	Air	0.65 (-1.69; 2.98)	.395	.051		
			Road	1.6 (-0.73; 3.93)	-	.395		
			Rail	2.72 (0.39; 5.05)	-	-		
Sex	F(1, 4942) = 0.1	.814	Male					
			Female					
				**Unstandardised regression coefficient (95% CI)**				
Baseline 30s ORP	F(1, 4942) = 1323.6	< .0001	-	0.84 (-32.33; -29.03)				
ORP at noise onset	F(1, 4942) = 75	< .0001	-	0.58 (3.86; 6.11)				
Time of night	F(1, 4942) = 4.2	.039	-	3.74 (0.37; 15,05)				
Night in study	F(1, 4942) = 1.2	.267	-	0.33 (-1.02; 0.28)				

EMM Estimated Marginal Means; CI Confidence Interval; df degrees of freedom.
[a] Covariates evaluated in the model at the following values: baseline 30s ORP=0.748; ORP at noise onset = 0.740; Time of night = 03:00; Night in study = 3.4
[b] Multiple testing corrections applied

Table S4 Sleep macrostructure estimated means and 95% confidence intervals. Data are adjusted for sex and time in study. P-values indicate unadjusted type III effects. Significant results after FDR adjustments are denoted with asterisks *** p<.001

Variable	Exposure condition				P-value
	Control	Pink only	Traffic only	Pink + Traffic	
Alpha intrusions EEG1 [1]	0.15 (-0.77; 1.07)	0.235 (-0.69; 1.16)	0.26 (-0.67; 1.18)	0.44 (-0.48; 1.36)	0.266
Alpha intrusions EEG2 [1]	0.36 (-0.50; 1.23)	0.30 (-0.56; 1.17)	0.41 (-0.45; 1.28)	0.68 (-0.19; 1.54)	0.024
Mean ORP wake	1.97 (1.90; 2.04)	2.03 (1.96; 2.10)	1.96 (1.92; 2.07)	2.00 (1.93; 2.07)	0.385
Mean ORP non-REM sleep	0.58 (0.50; 0.66)	0.64 (0.56; 0.72)	0.62 (0.54; 0.70)	0.63 (0.55; 0.71)	0.283
Mean ORP N3 sleep	0.33 (0.25; 0.41)	0.33 (0.24; 0.41)	0.34 (0.26; 0.43)	0.34 (0.26; 0.43)	0.971
Mean ORP total recording	0.74 (0.65; 0.82)	0.80 (0.72; 0.89)	0.80 (0.72; 0.89)	0.77 (0.68; 0.86)	0.141
Cumulative Sleep Index	854.8 (810.2; 899.4)	818.4 (773.9; 863.0)	825.4 (780.0; 870.8)	846.4 (801.9; 891.0)	0.097
Mean ORP Last 120 NREM / Wake	0.770 (0.61; 0.92)	0.88 (0.73; 1.04)	0.74 (0.58; 0.90)	0.74 (0.58; 0.89)	0.160
Min ORP First Half NREM	0.22 (0.17; 0.27)	0.21 (0.16; 0.26)	0.25 (0.20; 0.30)	0.20 (0.15; 0.25)	0.044
Wake time (minutes)	32.8 (17.8; 47.9)	40.4 (25.4; 55.4)	44.1 (28.8; 59.4)	36.1 (21.2; 51.1)	0.253
N1 Time (minutes)	46.1 (35.8; 56.4)	45.1 (34.8; 55.3)	52.1 (41.9; 63.0)	48.1 (38.1; 58.6)	0.443
N2 Time (minutes)	252.9 (237.8; 267.9)	253.7 (238.6; 268.8)	241.9 (226.3; 257.6)	264.1 (249.0; 279.2)	0.083
N3 Time (minutes)	61.2 (44.2; 78.3)	61.6 (44.5; 78.7)	55.7 (38.5; 73.0)	69.3 (52.2; 86.3)	0.077
REM Time (minutes)	91.3 (75.1; 107.5)	81.0 (64.8; 97.2)	93.3 (76.5; 110.0)	71.4 (55.2; 87.6)	0.053
TST (minutes)	451.9 (439.1; 464.8)	441.8 (429.0; 454.7)	442.8 (429.7; 455.8)	453.3 (440.5; 466.2)	0.017
N1 (% of TST)	10.2 (7.9; 12.5)	10.3 (7.9; 12.6)	12.0 (9.6; 14.3)	10.7 (8.4; 13.0)	0.385
N2 (% of TST)	56.1 (52.5; 59.6)	57.5 (54.0; 61.1)	54.8 (51.1; 58.4)	58.5 (54.9; 62.0)	0.136
N3 (% of TST)	13.5 (9.8; 17.2)	13.9 (10.2; 17.6)	12.4 (8.7; 16.2)	15.2 (11.5; 18.9)	0.101
REM (% of TST)	20.1 (16.7; 23.5)	18.2 (14.8; 21.6)	21.1 (17.5; 24.6)	15.7 (12.3; 19.1)	0.040
Sleep efficiency (%)	93.3 (90.3; 96.3)	91.7 (88.7; 94.7)	91.0 (87.9; 94.0)	92.7 (89.8; 95.7)	0.167
Total awakenings (n)	21.7 (16.2; 29.3)	24.0 (17.9; 30.1)	27.0 (20.8; 33.3)	23.7 (17.6; 29.8)	0.189
Total arousals (n)	134.2 (1116.2; 152.1) [a]	127.8 (109.8; 145.7) [bc]	145.9 (127.8; 164.1) [b]	158.3 (140.4; 176.3) [ac]	<0.00001***
Arousal+Awakening Index (n/h)	17.9 (15.2; 20.5) [de]	17.4 (14.7; 20.1) [fg]	19.9 (17.2; 22.5) [df]	21.1 (18.4; 23.8) [eg]	<0.00001***
ORP intrusions ≥1.75 EEG1 (n/h)	115.1 (92.5; 137.7)	118.8 (96.2; 141.4)	129.3 (106.2; 152.4)	119.0 (96.4; 141.6)	0.489
ORP intrusions ≥2.0 EEG1 (n/h)	85.4 (68.5; 102.4)	86.5 (69.9; 103.5)	96.1 (78.8; 113.4)	90.6 (73.7; 107.5)	0.466

ORP intrusions ≥1.75 EEG2 (n/h)	114.2 (77.8; 150.5)	140.9 (104.6; 177.3)	125.7 (87.9; 163.5)	123.7 (87.3; 160.1)	0.615
ORP intrusions ≥2.0 EEG2 (n/h)	85.4 (54.6; 116.2)	108.1 (77.3; 139.0)	91.6 (59.5; 123.7)	92.3 (61.4; 123.1)	0.627

[1] Log-transformed data

[a] Significant post-hoc difference between Control and Pink + Traffic conditions ($p < 0.001$)

[b] Significant post-hoc difference between Pink only and Traffic only conditions ($p = 0.005$)

[c] Significant post-hoc difference between Pink only and Pink + Traffic conditions ($p < 0.001$)

[d] Significant post-hoc difference between Control and Traffic only conditions ($p = 0.005$)

[e] Significant post-hoc difference between Control and Pink + Traffic conditions ($p < 0.001$)

[f] Significant post-hoc difference between Pink only and Traffic only conditions ($p < 0.001$)

[g] Significant post-hoc difference between Pink only and Pink + Traffic conditions ($p < 0.001$)

Table S5 Blood plasma metabolite estimated means and 95% confidence intervals. Data are adjusted for sex and time in study. P-values indicate unadjusted type III effects. Significant results after FDR adjustments are denoted with asterisks * p<.05

Metabolite	Exposure condition				P-value
	Control	Pink only	Traffic only	Pink + Traffic	
Ethanol (mmol/L)	Concentrations below detection limit				
Trimethylamine-N-oxide (mmol/L)	0.025 (0.015; 0.034)	0.029 (0.020; 0.039)	0.030 (0.021; 0.040)	0.025 (0.016; 0.034)	0.239
2-Aminobutyric acid (mmol/L)	Concentrations below detection limit				
Alanine (mmol/L)	0.242 (0.184; 0.299)	0.264 (0.207; 0.322)	0.267 (0.209; 0.325)	0.223 (0.165; 0.280)	0.351
Asparagine (mmol/L)	Concentrations below detection limit				
Creatine (mmol/L)	Concentrations below detection limit				
Creatinine (mmol/L)	0.084 (0.069; 0.099)	0.084 (0.069; 0.099)	0.094 (0.079; 0.109)	0.074 (0.059; 0.088)	0.280
Glutamic acid (mmol/L)	0.007 (0.001; 0.013)	0.002 (-0.004; 0.008)	0.009 (0.003; 0.015)	0.009 (0.003; 0.015)	0.225
Glycine (mmol/L)	0.210 (0.149; 0.272)	0.231 (0.169; 0.292)	0.239 (0.177; 0.300)	0.217 (0.155; 0.278)	0.566
Histidine (mmol/L)	0.066 (0.053; 0.08)	0.07 (0.056; 0.083)	0.079 (0.065; 0.092)	0.055 (0.042; 0.069)	0.076
Isoleucine (mmol/L)	0.045 (0.037; 0.053)	0.044 (0.036; 0.052)	0.050 (0.042; 0.059)	0.036 (0.027; 0.044)	0.036
Leucine (mmol/L)	0.091 (0.070; 0.111)	0.096 (0.075; 0.116)	0.115 (0.094; 0.135)	0.077 (0.057; 0.098)	0.005 *
Lysine (mmol/L)	0.111 (0.059; 0.163)	0.129 (0.077; 0.181)	0.091 (0.039; 0.142)	0.094 (0.042; 0.146)	0.624
Methionine (mmol/L)	0.050 (0.037; 0.063)	0.054 (0.041; 0.066)	0.065 (0.052; 0.078)	0.046 (0.033; 0.059)	0.070
N,N-Dimethylglycine (mmol/L)	0.002 (0.001; 0.002)	0.001 (0.001; 0.002)	0.001 (0.001; 0.002)	0.001 (0.0; 0.001)	0.035
Ornithine (mmol/L)	0.005 (0.0; 0.010)	0.005 (0.0; 0.010)	0.001 (-0.004; 0.006)	0.001 (-0.004; 0.006)	0.372
Phenylalanine (mmol/L)	0.027 (0.022; 0.031)	0.027 (0.022; 0.032)	0.028 (0.023; 0.033)	0.020 (0.015; 0.025)	0.087
Proline (mmol/L)	0.039 (-0.006; 0.084)	0.05 (0.006; 0.095)	0.049 (0.004; 0.094)	0.019 (-0.025; 0.064)	0.515
Sarcosine (mmol/L)	0.003 (0.001; 0.004)	0.003 (0.002; 0.005)	0.003 (0.001; 0.004)	0.003 (0.001; 0.004)	0.922
Threonine (mmol/L)	0.017 (-0.028; 0.062)	0.048 (0.004; 0.093)	0.062 (0.018; 0.107)	0.042 (-0.002; 0.087)	0.322
Tyrosine (mmol/L)	0.035 (0.029; 0.042)	0.035 (0.029; 0.041)	0.038 (0.032; 0.045)	0.029 (0.023; 0.035)	0.025
Valine (mmol/L)	0.190 (0.156; 0.225)	0.191 (0.156; 0.226)	0.209 (0.174; 0.244)	0.163 (0.128; 0.198)	0.122
2-Hydroxybutyric acid (mmol/L)	Concentrations below detection limit				
Acetic acid (mmol/L)	0.043 (0.03; 0.056)	0.041 (0.028; 0.054)	0.050 (0.037; 0.063)	0.046 (0.032; 0.059)	0.740
Citric acid (mmol/L)	Concentrations below detection limit				

Formic acid (mmol/L)	0.015 (0.011; 0.019)	0.016 (0.012; 0.020)	0.016 (0.012; 0.02)	0.015 (0.011; 0.019)	0.891
Lactic acid (mmol/L)	0.856 (0.613; 1.099)	0.891 (0.649; 1.134)	1.238 (0.995; 1.481)	0.773 (0.530; 1.016)	0.003 *
Succinic acid (mmol/L)	0.002 (0.001; 0.003)	0.002 (0.001; 0.003)	0.003 (0.002; 0.004)	0.002 (0.001; 0.003)	0.340
Choline (mmol/L)		Concentrations below detection limit			
2 Oxoglutaric acid (mmol/L)		Concentrations below detection limit			
3-Hydroxybutyric acid (mmol/L)	0.036 (0.002; 0.069)	0.042 (0.008; 0.075)	0.087 (0.054; 0.121)	0.023 (-0.011; 0.056)	0.017
Acetoacetic acid (mmol/L)	0.031 (0.023; 0.040)	0.027 (0.019; 0.035)	0.035 (0.027; 0.043)	0.019 (0.011; 0.027)	0.005 *
Acetone (mmol/L)	0.022 (0.010; 0.034)	0.028 (0.016; 0.04)	0.043 (0.031; 0.055)	0.02 (0.008; 0.032)	0.004 *
Pyruvic acid (mmol/L)	0.046 (0.033; 0.059)	0.045 (0.033; 0.058)	0.038 (0.026; 0.051)	0.044 (0.031; 0.057)	0.733
DGalactose (mmol/L)		Concentrations below detection limit			
Glucose (mmol/L)	4.305 (3.599; 5.012)	4.728 (4.022; 5.435)	4.744 (4.038; 5.450)	3.938 (3.232; 4.644)	0.291
Glycerol (mmol/L)	0.077 (0.042; 0.111)	0.023 (-0.011; 0.058)	0.051 (0.017; 0.086)	0.046 (0.012; 0.080)	0.140
Dimethylsulfone (mmol/L)	0.009 (0.006; 0.012)	0.010 (0.007; 0.014)	0.009 (0.006; 0.012)	0.008 (0.004; 0.011)	0.268
Ca-EDTA (mmol/L)	0.002 (0.001; 0.003)	0.003 (0.002; 0.005)	0.003 (0.001; 0.004)	0.002 (0.0; 0.003)	0.237
K-EDTA (mmol/L)	0.010 (0.008; 0.012)	0.012 (0.009; 0.014)	0.012 (0.010; 0.014)	0.009 (0.007; 0.011)	0.158

Table S6 Blood plasma metabolite estimated means and 95% confidence intervals. Data are adjusted for sex, time in study and EEG arousal and awakening index (n/h). P-values indicate unadjusted type III effects. Significant results after FDR adjustments are denoted with asterisks * p<.05; **p<.01

Metabolite	Exposure condition				Exposure P-value	EEG arousal & awakening index P-value
	Control	Pink only	Traffic only	Pink + Traffic		
Leucine (mmol/L)	0.095 (0.074; 0.116)	0.102 (0.080; 0.123)	0.115 (0.094; 0.136)	0.072 (0.051; 0.093)	0.001 **	0.068
Lactic acid (mmol/L)	0.907 (0.675; 1.139)	0.960 (0.723; 1.196)	1.187 (0.953; 1.422)	0.714 (0.481; 0.947)	0.006 *	0.064
Acetoacetic acid (mmol/L)	0.031 (0.023; 0.039)	0.027 (0.019; 0.035)	0.032 (0.024; 0.040)	0.019 (0.011; 0.027)	0.015 *	0.907
Acetone (mmol/L)	0.024 (0.012; 0.036)	0.030 (0.018; 0.043)	0.038 (0.026; 0.050)	0.019 (0.007; 0.031)	0.014 *	0.238

Table S7 Associations between EEG arousal and awakening index and blood plasma metabolite estimated. Data are adjusted for sex and time in study. P-values indicate unadjusted type III effects.

Metabolite	EEG arousal & awakening index (n/h)		
	Type III main effect	Coefficient (95% CI)	P-value
Leucine (mmol/L)	F(1,39) = 0.775,	0.001 (-0.002; 0.004)	.384
Lactic acid (mmol/L)	F(1,39) = 2.575	0.028 (-0.007; 0.062)	.117
Acetoacetic acid (mmol/L)	F(1,39) = 1.013	-0.001 (-0.002; 0.001)	.320
Acetone (mmol/L)	F(1,39) = 0.971	0.001 (-0.001; 0.003)	.331

Table S8 Morning questionnaire estimated means and 95% confidence intervals. Data are adjusted for sex and time in study. P-values for exposure indicate unadjusted type III effects. Significant results after FDR adjustments are denoted with asterisks * p<.05; ** p<.01; *** p<.001. Post-hoc tests were performed only when significant effects of exposure were found (Contrasts: C:P Control-Pink noise only; C:T Control-Traffic noise only; C:TP Control-Traffic + Pink noise; P:T Pink noise only-Traffic noise only; P:PT Pink noise only-Traffic + Pink noise; T:TP Traffic noise only-Traffic + Pink noise)

**Questionnaire item and scale**	**Control**	**Pink noise only**	**Traffic noise only**	**Traffic + Pink noise**	**Exposure p-value**	**Post-hoc p-values**					
						**C:P**	**C:T**	**C:TP**	**P:T**	**P:TP**	**T:TP**
Sleep quality, Very poor (0) to Very good (10)	7.2 (5.9; 8.5)	5.1 (3.8; 6.4)	5.0 (3.8; 6.3)	5.6 (4.3; 6.8)	.0163 *	.032	.032	.121	1.0	1.0	1.0
Sleep quality, 5-point verbal scale	3.3 (2.7; 3.9)	2.3 (1.7; 2.9)	2.3 (1.7; 2.9)	2.5 (1.9; 3.1)	.0234 *	.039	.051	0128	1.0	1.0	1.0
Alertness (KSS score 1-9)	4.5 (3.4; 5.7)	3.9 (2.7; 5.0)	3.0 (1.9; 4.2)	3.0 (1.9; 4.1)	.0105 *	.369	.023	.023	.354	.354	.971
Tired (0) to Rested (10)	5.8 (4.5; 7.1)	4.8 (3.5; 6.1)	4.7 (3.4; 6.0)	4.9 (3.6; 6.2)	.2917	-	-	-	-	-	-
Tense (0) to Relaxed (10)	7.3 (6.0; 8.5)	6.4 (5.1; 7.6)	5.9 (4.7; 7.2)	6.1 (4.9; 7.4)	.0825	-	-	-	-	-	-
Irritated (0) to Happy (10)	6.5 (5.3; 7.6)	5.6 (4.4; 6.8)	5.5 (4.3; 6.7)	5.5 (4.4; 6.7)	.5351	-	-	-	-	-	-
Estimated SOL (minutes)	21.6 (8.1; 35.2)	20.2 (6.6; 33.7)	29.6 (16.0; 43.1)	22 (8.4; 35.5)	.3792	-	-	-	-	-	-
Recalled awakenings (n)	2.1 (1.0; 3.2)	2.6 (1.4; 3.7)	2.9 (1.8; 4.1)	3.4 (2.3; 4.6)	.1412	-	-	-	-	-	-
Easy to sleep (0) to Difficult to sleep (10)	4.0 (2.6; 5.4)	5.0 (3.6; 6.5)	5.6 (4.1; 7)	4.9 (3.5; 6.4)	.0435	-	-	-	-	-	-
Slept better (0) to Slept worse (10) than usual	4.5 (3.2; 5.8)	5.6 (4.3; 7.0)	6.1 (4.8; 7.4)	6.3 (4.9; 7.6)	.0787	-	-	-	-	-	-
Sleep deep (0) to Slept light (10)	3.6 (2.3; 4.8)	5.6 (4.4; 6.9)	5.3 (4.0; 6.5)	5.5 (4.3; 6.8)	.0069 *	.013	.043	.019	1.0	1.0	1.0
Woke rarely (0) to Woke often (10)	4.4 (3.1; 5.7)	5.6 (4.3; 6.9)	6.1 (4.8; 7.4)	6.7 (5.4; 8.0)	.0303 *	.463	.141	.026	.909	.477	.909
Sleep disturbance by traffic noise (0-10)	0.4 (-1.3; 2.2)	3.4 (1.7; 5.2)	5.0 (3.3; 6.7)	5.7 (4.0; 7.5)	<.0001 ***	.022	.0004	<.0001	.262	.092	.482
Sleep disturbance by other noise (0-10)	1.0 (-0.8; 2.7)	3.8 (2.1; 5.6)	1.9 (0.2; 3.7)	4.4 (2.7; 6.2)	.0026 **	.024	.647	.006	.175	.647	.051
Noise caused poor sleep, 5-point verbal scale	0.2 (-0.5; 0.9)	1.6 (1.0; 2.3)	1.5 (0.8; 2.2)	2.0 (1.3; 2.7)	<.0001 ***	.0002	.0006	<.0001	.677	.586	.416
Noise caused awakenings, 5-point verbal scale	0.0 (-0.7; 0.6)	1.8 (1.1; 2.4)	1.8 (1.1; 2.4)	2.2 (1.5; 2.8)	<.0001 ***	.0002	.0002	<.0001	.932	.820	.820
Noise caused difficulty sleeping, 5-point verbal scale	0.1 (-0.6; 0.8)	1.3 (0.6; 2.0)	1.6 (0.9; 2.3)	1.5 (0.9; 2.2)	<.0001 ***	.0006	<.0001	<.0001	.893	.893	.893
Noise caused tiredness in morning, 5-point verbal scale	0.2 (-0.6; 1.0)	1.8 (1.0; 2.6)	1.9 (1.0; 2.7)	1.6 (0.8; 2.4)	.0002 ***	.0009	.0009	.004	1.0	1.0	1.0
Sound-induced auditory fatigue (5-point verbal scale)	0.0 (-0.6; 0.5)	1.0 (0.4; 1.5)	0.8 (0.2; 1.3)	0.8 (0.2; 1.3)	.0022 **	.003	.019	.019	1.0	1.0	1.0

Table S9 Evening questionnaire estimated means and 95% confidence intervals. Data are adjusted for sex and time in study. P-values indicate unadjusted type III effects.

Questionnaire item and scale	Control	Pink noise only	Traffic noise only	Traffic + Pink noise	Exposure p-value
Alertness (KSS score 1-9)	1,9 (1,2; 2,6)	1,9 (1,1; 2,6)	1,7 (1.0; 2,4)	2,4 (1,6; 3,2)	.4978
Tired (0) to Rested (10)	Model could not converge				
Tense (0) to Relaxed (10)	6,2 (4,5; 8.0)	6.0 (4,2; 7,8)	6,7 (5,1; 8,4)	5,8 (4.0; 7,6)	.6712
Irritated (0) to Happy (10)	6,7 (5,2; 8,2)	6,5 (5.0; 8,1)	6,3 (4,9; 7,7)	5,9 (4,4; 7,5)	.8304
Sound-induced auditory fatigue (5-point verbal scale)	Model could not converge				

Table S10 Morning cognition test estimated means (z-scored) and 95% confidence intervals for each speed and accuracy outcome. Data are adjusted for sex and time in study. P-values indicate unadjusted type III effects. No significant effects survived adjustment for multiple testing (FDR).

Outcome (morning testing)		Estimated means (95% CI)				Exposure p-value
		**Control**	**Pink noise only**	**Traffic noise only**	**Traffic + Pink noise**	
Speed	MP	0.01 (-0.72; 0.75)	0.16 (-0.57; 0.90)	-0.40 (-1.14; 0.33)	-0.50 (-1.24; 0.23)	.388
	VOLT	-0.34 (-0.89; 0.21)	-0.37 (-0.92; 0.18)	-0.29 (-0.84; 0.26)	0.41 (-0.14; 0.96)	.037
	F2B	-0.24 (-0.90; 0.42)	0.21 (-0.45; 0.87)	-0.06 (-0.72; 0.59)	0.24 (-0.42; 0.90)	.312
	AM	0.08 (-0.55; 0.71)	-0.23 (-0.86; 0.40)	-0.03 (-0.66; 0.60)	-0.12 (-0.75; 0.50)	.701
	LOT	-0.04 (-0.70; 0.62)	-0.13 (-0.79; 0.53)	-0.07 (-0.72; 0.59)	-0.22 (-0.88; 0.44)	.900
	ERT	-0.19 (-0.84; 0.46)	0.01 (-0.64; 0.66)	-0.06 (-0.71; 0.60)	0.27 (-0.39; 0.92)	.211
	MRT	-0.16 (-0.81; 0.50)	0.21 (-0.44; 0.87)	0.08 (-0.58; 0.73)	0.13 (-0.52; 0.79)	.499
	DSST	0.12 (-0.47; 0.71)	0.27 (-0.32; 0.86)	0.26 (-0.33; 0.85)	-0.09 (-0.67; 0.50)	.517
	BART	-0.07 (-0.72; 0.59)	-0.09 (-0.74; 0.56)	0.04 (-0.61; 0.70)	0.08 (-0.57; 0.74)	.756
	PVT	0.20 (-0.39; 0.79)	-0.56 (-1.14; 0.03)	0.06 (-0.53; 0.65)	-0.06 (-0.65; 0.52)	.112
	Composite	-0.03 (-0.39; 0.33)	-0.03 (-0.39; 0.33)	-0.02 (-0.38; 0.34)	-0.02 (-0.38; 0.34)	.997
Accuracy	MP	-0.34 (-0.89; 0.21)	-0.37 (-0.92; 0.18)	-0.29 (-0.84; 0.26)	0.41 (-0.14; 0.96)	.037
	VOLT	0.08 (-0.55; 0.71)	-0.23 (-0.86; 0.40)	-0.03 (-0.66; 0.60)	-0.12 (-0.75; 0.50)	.701
	F2B	-0.19 (-0.84; 0.46)	0.01 (-0.64; 0.66)	-0.06 (-0.71; 0.60)	0.27 (-0.39; 0.92)	.211
	AM	0.12 (-0.47; 0.71)	0.27 (-0.32; 0.86)	0.26 (-0.33; 0.85)	-0.09 (-0.67; 0.50)	.517
	LOT	0.20 (-0.39; 0.79)	-0.56 (-1.14; 0.03)	0.06 (-0.53; 0.65)	-0.06 (-0.65; 0.52)	.112
	ERT	0.37 (-0.16; 0.91)	0.14 (-0.40; 0.68)	0.13 (-0.41; 0.66)	-0.11 (-0.65; 0.43)	.649
	MRT	0.0 (-0.57; 0.56)	-0.05 (-0.62; 0.51)	0.11 (-0.46; 0.67)	0.13 (-0.44; 0.70)	.885
	DSST	0.14 (-0.52; 0.80)	-0.21 (-0.88; 0.45)	-0.17 (-0.84; 0.49)	-0.29 (-0.95; 0.38)	.779
	BART [a]	0.03 (-0.58; 0.64)	-0.03 (-0.64; 0.58)	-0.29 (-0.90; 0.32)	0.0 (-0.61; 0.61)	.463
	PVT	-0.13 (-0.81; 0.55)	-0.12 (-0.80; 0.56)	-0.28 (-0.96; 0.40)	-0.21 (-0.89; 0.47)	.815
	Composite	0.04 (-0.26; 0.35)	-0.11 (-0.42; 0.19)	-0.03 (-0.34; 0.27)	-0.01 (-0.31; 0.30)	.396

[a] Data for the BART indicate risk taking rather than accuracy

Table S11 Evening cognition test estimated means (z-scored) and 95% confidence intervals for each speed and accuracy outcome. Data are adjusted for sex and time in study. P-values indicate unadjusted type III effects.

Outcome (evening testing)		Estimated means (95% CI)				Exposure p-value
		Control	Pink noise only	Traffic noise only	Traffic + Pink noise	
Speed	MP	-0.08 (-0.63; 0.46)	-0.15 (-0.76; 0.45)	0.38 (-0.15; 0.92)	0.42 (-0.18; 1.03)	.249
	VOLT	0.34 (-0.30; 0.98)	-0.07 (-0.78; 0.64)	0.07 (-0.56; 0.70)	-0.17 (-0.89; 0.54)	.641
	F2B	0.37 (-0.04; 0.78)	0.42 (-0.01; 0.85)	0.21 (-0.20; 0.61)	0.49 (0.06; 0.93)	.435
	AM	0.09 (-0.59; 0.77)	0.41 (-0.31; 1.14)	-0.11 (-0.79; 0.56)	0.07 (-0.66; 0.80)	.487
	LOT	-0.02 (-0.57; 0.54)	0.15 (-0.46; 0.76)	0.51 (-0.04; 1.06)	0.17 (-0.44; 0.78)	.412
	ERT	0.23 (-0.48; 0.94)	0.16 (-0.61; 0.93)	-0.12 (-0.83; 0.58)	-0.13 (-0.90; 0.64)	.694
	MRT	0.17 (-0.38; 0.73)	0.22 (-0.36; 0.81)	0.40 (-0.16; 0.95)	0.27 (-0.31; 0.86)	.726
	DSST	-0.09 (-0.61; 0.43)	0.13 (-0.44; 0.71)	0.34 (-0.17; 0.85)	-0.23 (-0.81; 0.35)	.355
	BART	0.23 (-0.33; 0.79)	0.54 (-0.05; 1.13)	0.39 (-0.17; 0.94)	0.31 (-0.27; 0.90)	.596
	PVT	0.09 (-0.61; 0.79)	-0.25 (-1.02; 0.53)	0.11 (-0.59; 0.80)	-0.56 (-1.33; 0.22)	.394
	Composite	0.12 (-0.18; 0.42)	0.14 (-0.17; 0.45)	0.28 (-0.02; 0.58)	0.31 (0.01; 0.62)	.084
Accuracy	MP	0.34 (-0.30; 0.98)	-0.07 (-0.78; 0.64)	0.07 (-0.56; 0.70)	-0.17 (-0.89; 0.54)	.641
	VOLT	0.09 (-0.59; 0.77)	0.41 (-0.31; 1.14)	-0.11 (-0.79; 0.56)	0.07 (-0.66; 0.80)	.487
	F2B	0.23 (-0.48; 0.94)	0.16 (-0.61; 0.93)	-0.12 (-0.83; 0.58)	-0.13 (-0.90; 0.64)	.694
	AM	-0.09 (-0.61; 0.43)	0.13 (-0.44; 0.71)	0.34 (-0.17; 0.85)	-0.23 (-0.81; 0.35)	.355
	LOT	0.09 (-0.61; 0.79)	-0.25 (-1.02; 0.53)	0.11 (-0.59; 0.80)	-0.56 (-1.33; 0.22)	.394
	ERT	-0.28 (-0.98; 0.42)	0.08 (-0.68; 0.85)	-0.02 (-0.71; 0.67)	0.29 (-0.48; 1.06)	.576
	MRT	-0.33 (-1.13; 0.47)	0.0 (-0.88; 0.88)	-0.44 (-1.23; 0.35)	0.09 (-0.80; 0.97)	.653
	DSST	-0.06 (-0.71; 0.59)	0.32 (-0.39; 1.03)	0.57 (-0.08; 1.21)	-0.16 (-0.87; 0.55)	.189
	BART [a]	0.07 (-0.54; 0.68)	-0.16 (-0.81; 0.49)	-0.13 (-0.73; 0.48)	-0.14 (-0.79; 0.51)	.838
	PVT	0.15 (-0.45; 0.75)	0.11 (-0.53; 0.74)	-0.20 (-0.79; 0.40)	0.11 (-0.53; 0.74)	.483
	Composite	0.01 (-0.30; 0.32)	0.11 (-0.23; 0.44)	0.02 (-0.29; 0.33)	-0.10 (-0.43; 0.23)	.646

[a] Data for the BART indicate risk taking rather than accuracy

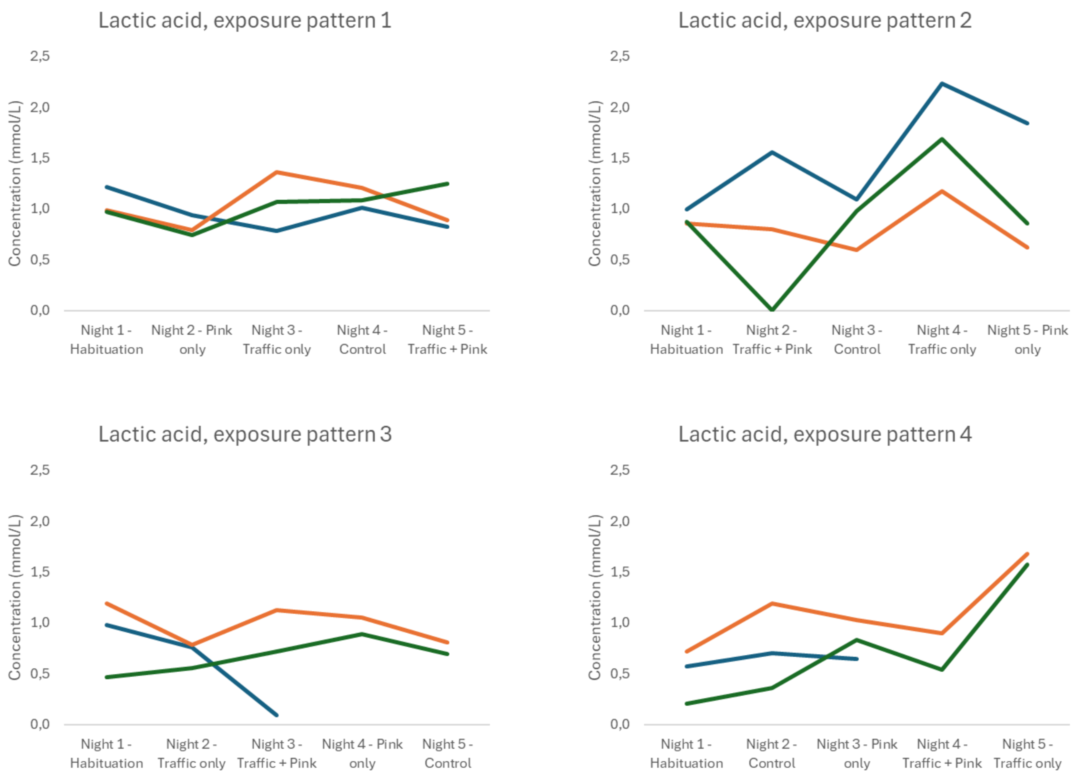

Figure S2 Time series of lactic acid concentrations. Subplots are grouped according to the exposure pattern, i.e. the presentation order, of the randomised exposure nights. Each line represents single daily samples from one study participant.

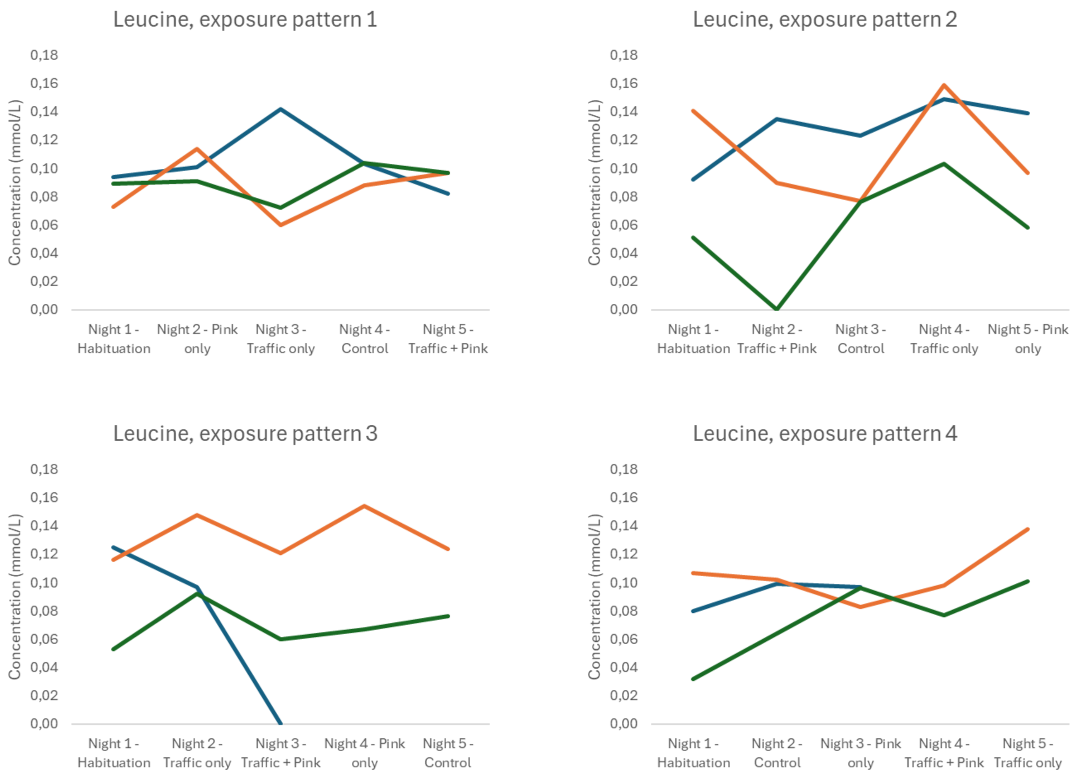

Figure S3 Time series of leucine concentrations. Subplots are grouped according to the exposure pattern, i.e. the presentation order, of the randomised exposure nights. Each line represents single daily samples from one study participant.

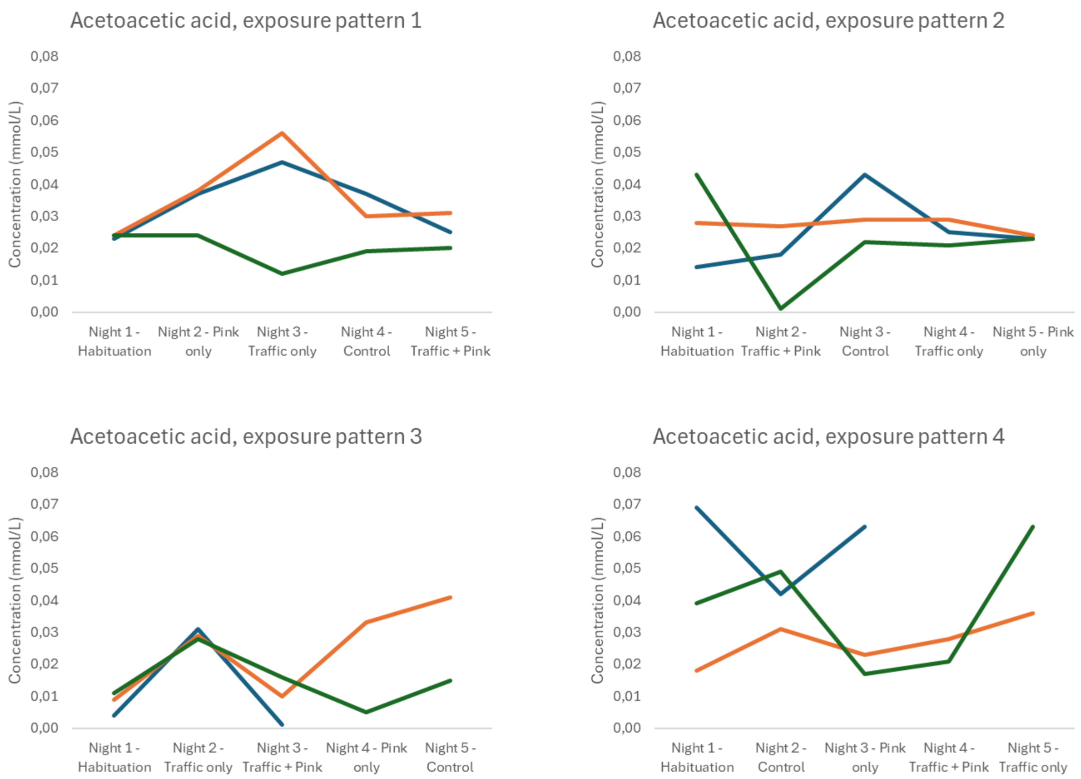

Figure S4 Time series of acetoacetic concentrations. Subplots are grouped according to the exposure pattern, i.e. the presentation order, of the randomised exposure nights. Each line represents single daily samples from one study participant.

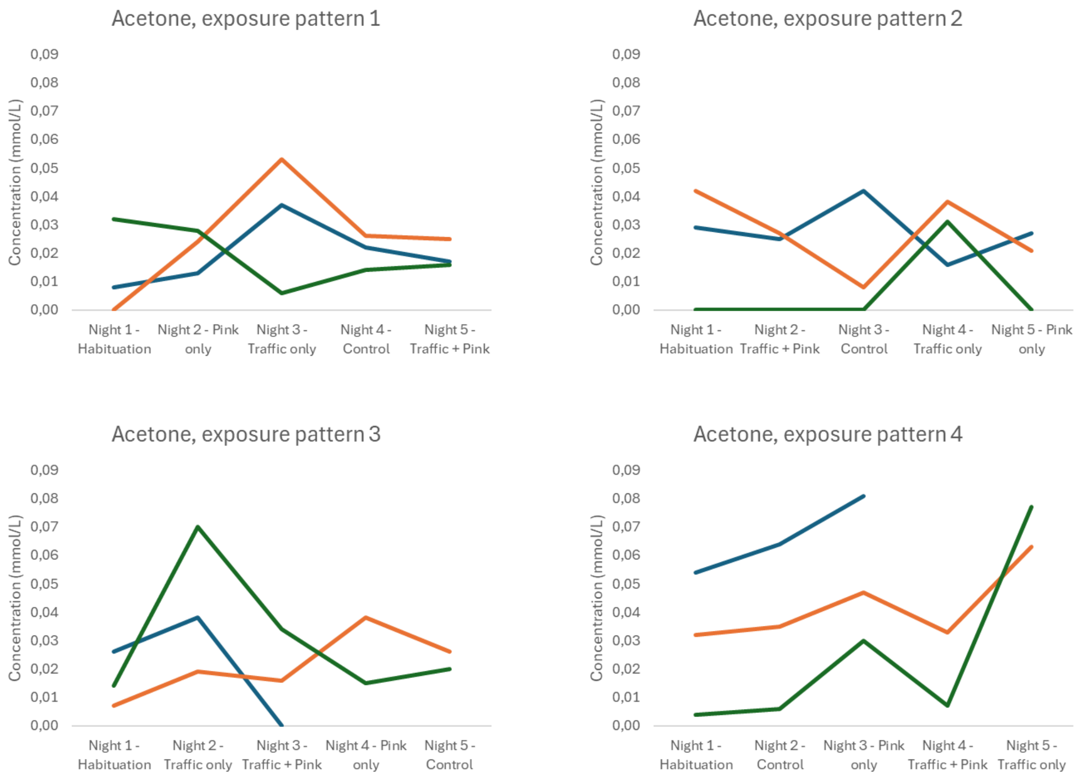

Figure S5 Time series of acetone concentrations. Subplots are grouped according to the exposure pattern, i.e. the presentation order, of the randomised exposure nights. Each line represents single daily samples from one study participant.

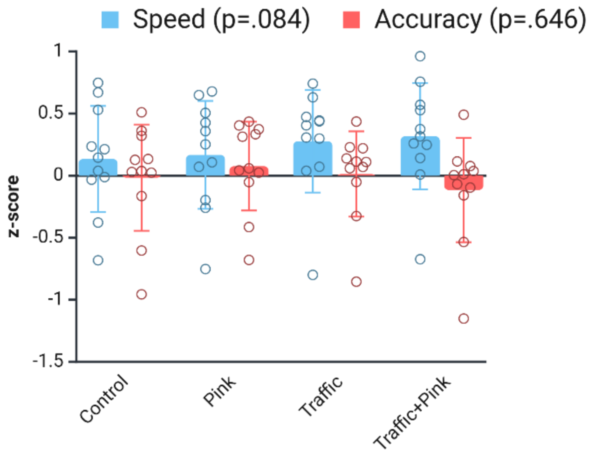

Figure S6 Individual cognition performance scores for morning test administrations (columns and error bars indicate mean ±SD). Point estimates and p-values are results of GLMM adjusted for sex and time in study.
