# Supplementary material for: Pink noise reduces impact of traffic noise on sleep and the blood metabolome: a cross-over pilot study
Source: Commun Med (Lond). 2026 Jan 10;6:114. doi: 10.1038/s43856-026-01380-5 (PMC12901012; doi:10.1038/s43856-026-01380-5)
Supplement: Supplementary file 2 — Description of Additional Supplementary Files [file 43856_2026_1380_MOESM2_ESM.pdf]

Description of Additional Supplementary Files

File name: Supplementary Data 1

Description: Source data underlying the main figures of this paper
